# TIR1 auxin receptors are implicated in the differential response to 4-Cl-IAA and IAA in developing pea fruit

**DOI:** 10.1093/jxb/ery456

**Published:** 2019-01-30

**Authors:** Charitha P A Jayasinghege, Jocelyn A Ozga, Courtney D Nadeau, Harleen Kaur, Dennis M Reinecke

**Affiliations:** Plant BioSystems, Department of Agricultural, Food and Nutritional Science University of Alberta, Edmonton, Alberta, Canada

**Keywords:** Auxin, auxin receptors, 4-chloroindole-3-acetic acid, fruit development, hormonal interaction, indole-3-acetic acid, *Pisum sativum*, TIR1/AFB genes

## Abstract

The auxins indole-3-acetic acid (IAA) and 4-chloroindole-3-acetic acid (4-Cl-IAA) occur naturally in pea (*Pisum sativum*); however, only 4-Cl-IAA mimics the presence of seeds in stimulating pericarp growth. To examine if this differential auxin effect is mediated through TIR1/AFB auxin receptors, pea *TIR1* and *AFB2* homologs were functionally characterized in Arabidopsis, and receptor expression, and auxin distribution and action were profiled in developing pea fruits. *PsTIR1a*, *PsTIR1b*, and *PsAFB2* restored the auxin-sensitive root growth response to the mutant Arabidopsis seedlings *Attir1-10* and/or *Attir1-10 afb2-3*. Expression of *PsTIR1* or *AtTIR1* in *Attir1-10 afb2-3* mutants also restored the greater root inhibitory response of 4-Cl-IAA compared to that of IAA, implicating TIR1 receptors in this response. The ability of 4-Cl-IAA to stimulate a stronger *DR5::GUS* auxin response than IAA at the same concentration in pea pericarps was associated with its ability to enrich the auxin-receptor transcript pool with *PsTIR1a* and *PsAFB2* by decreasing the transcript abundance of *PsTIR1b* (mimicking results in pericarps with developing seeds). Therefore, the markedly different effect of IAA and 4-Cl-IAA on pea fruit growth may at least partially involve TIR1/AFB receptors and the differential modulation of their population, resulting in specific Aux/IAA protein degradation that leads to an auxin-specific tissue response.

## Teaser

The pea auxin receptors PsTIR1a, PsTIR1b, and PsAFB2 are involved in auxin perception, and the TIR1s are implicated in the differential growth response to 4-Cl-IAA and IAA in pea fruit.

## Introduction

Auxins are major regulators of plant reproductive and vegetative development. Auxins occurring naturally in plants include the most ubiquitous auxin indole-3-acetic acid (IAA), indole-3-butyric acid, phenylacetic acid (PAA, a weak auxin), and the chlorinated auxin 4-chloroindole-3-acetic acid (4-Cl-IAA; Reinecke *et al.*, 1999). 4-Cl-IAA is a potent auxin tested in many different bioassays and reported to be 1.3–50 times more active than IAA (Reinecke, 1999). To date, species known to synthesize 4-Cl-IAA are restricted to the phylogenetic clades of the Fabeae and Trifoleae in the Fabaceae family, which includes *Pisum sativum* (pea), and it is found in high concentrations in the seeds of those plants (Reinecke, 1999; Lam *et al.*, 2015). How different forms of natural auxins contribute to the overall auxin function in plants remains unclear. However, 4-Cl-IAA has been recognized as a regulator of fruit (Ozga and Reinecke, 2003; Ozga *et al.*, 2009) and seed development in pea (McAdam *et al.*, 2017).

The presence of developing seeds within the fruit is a requirement for normal ovary development in most flowering plants, including pea. The lack of ovule fertilization or seed removal results in ovary (pericarp) senescence and subsequent pea fruit abscission (Ozga *et al.*, 1992; Ozga and Reinecke, 1999). Application of 4-Cl-IAA to the deseeded pericarp stimulates growth; in contrast, application of the other naturally occurring auxin IAA does not (Reinecke *et al.*, 1995). A major determinant of 4-Cl-IAA-specific pericarp growth promotion is its ability to stimulate gibberellin (GA) biosynthesis (van Huizen *et al.*, 1995, 1997; Ozga *et al.*, 2003, 2009). In other species, such as tomato (*Solanum lycopersicum*; Serrani *et al.*, 2008) and Arabidopsis (Dorcey *et al.*, 2009), IAA stimulates fruit growth via GAs. Furthermore, in pea 4-Cl-IAA, but not IAA, also inhibits the ethylene response in deseeded pericarps, potentially via the inhibition of ethylene signaling (Johnstone *et al.*, 2005; Jayasinghege *et al.*, 2017), enhancing the promotion of pericarp growth. These differential interactions of IAA and 4-Cl-IAA with the GA and ethylene biosynthesis and signaling pathways likely play a primary role in determining the effects of the two auxins on pea pericarp development. However, the underlying signaling mechanisms that lead to these differential auxin effects on the hormonal pathways within the fruit are not known.

Repeated auxin application and dose–response experiments have demonstrated that the comparatively lower activity of IAA, or its lower chemical stability compared to 4-Cl-IAA, is not likely to be the primary reason for its inability to stimulate pea pericarp growth (Reinecke *et al.*, 1995; Reinecke, 1999). Auxin analogs with varying substitutions at the fourth position of the indole ring, or analogs with chlorine or fluorine substituents at different positions of the indole ring, have been evaluated for their ability to induce pericarp development. These studies have shown that the position of the substituent on the indole ring, its size, and its lipophilicity are important factors in determining the activity of the analog, with the fourth position on the ring and a substituent size of approximately that of a chlorine atom being optimal for biological activity with respect to stimulating pea pericarp growth (Reinecke *et al.*, 1995, 1999). Based on these observations, it has been speculated that the differential effects of IAA and 4-Cl-IAA on pea pericarp development may rest at the auxin-receptor level (Reinecke *et al.*, 1999).

The TRANSPORT INHIBITOR RESPONSE 1/AUXIN SIGNALING F-BOX (TIR1/AFB) and AUXIN/INDOLE-3-ACETIC ACID (Aux/IAA) protein families act as co-receptors in auxin signaling. The interaction of the co-receptors in the presence of auxin initiates the proteasome-mediated degradation of Aux/IAAs and subsequent activation of the auxin response (Calderón Villalobos *et al.*, 2012). The auxin-dependent interactions of the TIR1/AFB and Aux/IAA proteins appear to depend on which TIR1/AFB and Aux/IAA proteins combine to make the co-receptor, which type of auxin is present, and on the auxin concentration (Calderón Villalobos *et al.*, 2012; Havens *et al.*, 2012). For instance, the 50% effective concentration (EC_50_) of auxin required for the degradation of Arabidopsis Aux/IAAs differs depending on the Aux/IAA protein and F-box protein (TIR1 or AFB2) complex. The EC_50_ values are also higher with the weak auxin PAA compared to that of IAA or 4-Cl-IAA within a specific Aux/IAA protein and F-box protein complex (Shimizu-Mitao and Kakimoto, 2014).

The TIR1/AFB auxin-receptor protein family is conserved across land plants and phylogenetic analysis divides the family into four clades: TIR1, AFB2, AFB4, and AFB6. Arabidopsis contains three TIR1/AFB clades with two alleles per clade (*AtTIR1* and *AtAFB1*; *AtAFB2* and *AtAFB3*; *AtAFB4* and *AtAFB5*; Parry *et al.*, 2009). Pea contains TIR1/AFB gene members from four clades (TIR1a and TIR1b; AFB2; AFB4; AFB6; Ozga *et al.*, 2010; Ligerot *et al.*, 2017). Much of our current understanding of auxin-receptor function comes from the auxin response in Arabidopsis root growth assays. In these assays, the AtTIR1 and AtAFB1 proteins do not contribute equally to auxin signaling that affects growth, with a functional AtTIR1 being sufficient for normal auxin response in the absence of *AtAFB1* (Parry *et al.*, 2009). Although the absence of a functional AtTIR1 (*Attir1-1* mutant) shows the highest auxin insensitivity in the Arabidopsis root elongation assays, the *Attir1-1 Atafb2-3* double-mutant further enhances auxin insensitivity, demonstrating that AFB2 also has a role in root growth (Parry *et al.*, 2009). While TIR1 followed by AFB2 are likely the most prominent auxin receptors regulating Arabidopsis root elongation, much less is known about the role of these receptors in Arabidopsis fruit development. The Arabidopsis *tir1* and *tir1afb2* mutants generally produce fruits similar to that of the wild-type (Dharmasiri *et al.*, 2005); however, treatment with the auxin transport inhibitor *N*-1-naphthylphthalamic acid induces a greater frequency of reduced-valve and valveless silique phenotypes in these mutants compared with the wild-type (Ståldal *et al.*, 2008; Zúñiga-Mayo *et al.*, 2014), supporting a role of these auxin receptors in auxin-related fruit developmental processes. Little is known about the distinct functions of auxin receptors in species other than Arabidopsis due to the lack of available auxin-receptor mutants. Overexpression of auxin-receptor genes in tomato, including *SlTIR1* (Ren *et al.*, 2011) and *CsTIR1* or *CsAFB2* from cucumber (*Cucumis sativus*) (Xu *et al.*, 2017), is reported to induce parthenocarpic fruit formation, showing that increasing ovary auxin sensitivity by elevating auxin-receptor abundance can modify tomato fruit development. To date, the only auxin-receptor mutant characterized in pea is an AFB4 mutant (also named PsAFB4/5 or *ramosus2*), which has been shown to be involved in the regulation of shoot branching (Ligerot *et al.*, 2017).

In this study, in order to understand auxin perception in pea, the pea TIR1/AFB2 homologs were functionally characterized in Arabidopsis auxin-receptor mutant backgrounds to evaluate their role in the perception of 2,4-D. The TIR1 homologs were further tested for their perception of IAA and 4-Cl-IAA to determine whether they contribute to the differential action of IAA and 4-Cl-IAA in pea fruit development. The regulation of expression of pea pericarp auxin receptor genes by seeds and/or auxin was assessed to determine whether this could be a mechanism to modulate pericarp auxin responsiveness during fruit set. The expression of an auxin-responsive reporter gene (*DR5::GUS*) was also evaluated to determine whether modulation of auxin-receptor gene expression and/or auxin signaling are possible mechanisms involved in the differential action of IAA and 4-Cl-IAA during fruit development.

## Materials and methods

### Plant materials

The pea cultivar *Pisum sativum* L. cv. I_3_ (Alaska-type) was used for all pea studies. Plants were grown in a growth chamber with a 16/8 h light/dark photoperiod at 19/17 °C as described by Jayasinghege *et al.* (2017).

For the fruit developmental study, pistils (pericarp plus stigma and style; hereafter referred to as fruits) were used at the given developmental stage, located at flowering nodes 1–6 and within a specific pericarp length range (1 d after anthesis, DAA: 8–12 mm; 2 DAA: 15–20 mm; 3 DAA: 26–33 mm) and/or mean seed weight range (5 DAA: 1.2–2.5 mg; 6 DAA: 5–7 mg; 7 DAA: 14–16 mg; 8 DAA: 20–28 mg; 10 DAA: 70–100 mg). For non-pollinated fruits, floral buds at –2 DAA were emasculated, and tissues were collected at –2, 0, 1, 2, and 3 DAA. Fruits were collected onto ice and immediately dissected into seed/ovule, pericarp wall, pericarp dorsal vascular suture, and pericarp ventral vascular suture tissues (see Supplementary Fig. S1A at *JXB* online), except for those at –2 DAA, where the ovules were removed and the fruits were harvested.

For hormonal treatments, fruits at 2 DAA measuring 15–20 mm in length were split, deseeded, and treated with IAA or 4-Cl-IAA (50 μM in 0.1% aqueous Tween 80), ethephon (ethylene-releasing agent; 1000 mg l^–1^ in 0.1% aqueous Tween 80), or silver thiosulfate (STS, inhibitor of ethylene action; 1 mM in 0.1% aqueous Tween 80) either alone or in combination. Auxin or auxin–ethephon combinations were IAA plus 4-Cl-IAA, IAA plus ethephon, 4-Cl-IAA plus ethephon, all in 0.1% aqueous Tween 80. The split (split pericarps with seeds, SP) and split and deseeded (split pericarp, no seeds, SPNS) controls were treated with 0.1% aqueous Tween 80. All hormonal and control treatments were applied 12 h after pericarp splitting and seed removal, with one exception: STS was applied to the pericarp immediately after splitting and deseeding, with subsequent hormonal application occurring 12 h after STS application. Solutions (30 μl) were applied to the inside surface of the pericarp wall (endocarp), and the pericarps were attached to the plant throughout the experiment. Samples were collected into liquid nitrogen at 0, 2, 8, and 12 h after solution application (12, 14, 20, and 24 h after pericarp splitting, splitting and deseeding, or deseeding and STS treatment) and stored at –80 °C.

The *DR5::GUS* construct in the pRD400 vector (DeMason and Polowick, 2009) was transformed into pea as described by Reinecke *et al.* (2013), and T_3_ generation homozygous plants were studied. DR5-driven expression of the GUS (β-glucuronidase) marker gene was monitored over the course of development in pre-pollinated (–2 DAA), and pollinated fruits at 0, 3, 5, 8, and 10 DAA, and in 2 DAA deseeded pericarps treated with auxin.

For hormone quantification, non-pollinated fruits (ovules removed) at 3 DAA, pericarps from pollinated fruits (seeds removed) at 0, 3, 5, and 8 DAA, pericarp tissues from pollinated fruits (central wall, ventral vascular suture, dorsal vascular suture) at 5 DAA and 8 DAA (funiculus removed), and seeds at 8 DAA were harvested, immediately frozen in liquid nitrogen, and stored at –80 °C.

For gene expression analysis in different vegetative organs, tissues of 12-d-old pea seedlings were used. The plants had six or seven nodes below the shoot apex, and the cotyledonary node was designated as node 1. Leaves attached to the fourth node were collected as mature leaves (mean length 31±2 mm) and leaves attached to the sixth or seventh nodes were collected as immature leaves (mean length 8±2 mm). Internodes between nodes 3 and 4 were collected as mature tissues (mean length 25±5 mm) and the most apical internodes (between nodes 5 and 6, or 6 and 7) were collected as immature internodes (mean length 4±2 mm). The shoot apices were also collected from all the seedlings. For the auxin treatment of seedlings, seeds were germinated and grown in Magenta GA-7 vessels (Sigma-Aldrich) at room temperature (~22 °C) in continuous darkness. At 2 d after imbibition (DAI), water-grown seedlings were transferred to either water, IAA (1 µM), or 4-Cl-IAA (1 µM) and grown for an additional 2 d prior to harvesting of the plumule, epicotyl, and root-tip (6–7 mm) at 4 DAI. All seedling tissue samples were immediately frozen in liquid nitrogen and stored at –80 °C until RNA extraction.

### Identification of pea TIR1/AFB family members

Five putative TIR1/AFB family members in the pea genome were identified by screening a small-scale Roche 454 Titanium sequencing database derived from the seed coats of the following pea cultivars at 10 DAA: I_3_ (Alaska-type), Courier, Canstar, Solido, and LAN 3017 (Ferraro, 2014). Complete coding sequences (CDSs) of all the genes were amplified from total RNA (primer sequences listed in Supplementary Table S1), cloned into the pCR8 vectors (Invitrogen), and verified by Sanger sequencing. Sequence comparisons and construction of a phylogenetic tree by the neighbor-joining method were conducted using the PRALINE sequence alignment program and the MEGA X program, respectively (Supplementary Protocol S1).

### Gene expression analysis

RNA was isolated using a modified TRIzol (Life Technologies) method (Ayele *et al.*, 2006). qRT-PCR primers, probes, and their reaction efficiencies are listed in Supplementary Table S2. All the probes were double-quenched and contained Iowa Black FQ (IBFQ) quencher at the 3′-end and 6-FAM fluorescent dye at the 5′-end (Integrated DNA Technologies). The only exception was the 18S rRNA control, which contained the fluorescent reporter VIC and the quencher TAMRA (Ozga *et al.*, 2003). The CDSs of *PsTIR1a* and *PsTIR1b* share 82% identity; therefore, the target specificity of the qRT-PCR primers was confirmed by sequencing the PCR products. A TaqMan One-Step RT-PCR Master-Mix Reagents Kit or RNA-to-Ct 1-Step Kit (Applied Biosystems) was used for qRT-PCR analysis. The reactions were performed in a StepOnePlus Real-Time PCR system as described by Jayasinghege *et al.* (2017; Supplementary Protocol S2).

### Transgenic Arabidopsis plants and root growth analyses

All Arabidopsis lines were from the Columbia (Col-0) ecotype. Wild-type Col-0 (CS 70000) and the T-DNA insertion mutants *Attir1-10* (SALK_090445C) and *Atafb2-3* (SALK_137151) were obtained from the Arabidopsis Biological Resource Center (https://abrc.osu.edu/). Backgrounds of the mutants were cleaned by backcrossing once with Col-0. Background-cleaned *Atafb2-3* and *Attir1-10* lines were crossed to obtain double-mutants. Transformation vectors were created by placing the *PsTIR1a*, *PsTIR1b*, *PsAFB2* or *AtTIR1* CDSs following the *AtTIR1* 3-kb promoter region (–1 bp to –3008 bp region from the start codon) into a modified version of pCAMBIA1300 named pCm1300-polyA (provided by Dr Enrico Scarpella, University of Alberta). Arabidopsis floral dip transformation was performed as described by Zhang *et al.* (2006).

Root growth assays were adapted from Parry *et al.* (2009). Transgenic lines homozygous for the transgene insert that expressed the transgene at reliable levels were selected for the assays (Supplementary Fig. S1). Seedlings were grown vertically under continuous light and aseptic conditions. Uniformly-sized seedlings at 4 d old were transferred aseptically to media with or without auxin (Arabidopsis lines expressing *PsTIR1a*, *PsTIR1b*, and *AtTIR1* at 50 nM and 70 nM 2,4-D, or 400 nM and 800 nM IAA or 4-Cl-IAA; *PsAFB2* at 70 nM and 90 nM 2,4-D) for an additional 3 d. The root elongation of each genotype in auxin medium is expressed as a percentage of the same genotype in the medium without auxin (Supplementary Protocol S3).

### *DR5::GUS* expression analysis

For GUS staining, cross-sections of fresh tissue (<1 mm thick) of the mid-pericarp region from *DR5::GUS* expressing lines were made by hand using a scalpel. The staining procedure was adapted from Hull and Devic (1995). The sections were submerged in GUS staining solution (Supplementary Protocol S4) and placed in a vacuum desiccator for 30 min and then in an incubator at 37 °C for 12 h in darkness. After washing with 70% ethanol, the tissue sections were observed under a stereo microscope. To verify that the staining observed in the transgenic plants was due to the expression of *DR5::GUS*, SPNS pericarps treated with IAA, 4-Cl-IAA, or 0.1% aqueous Tween 80 were also examined in wild-type plants and no GUS staining was detected in the tissue 8 h after the hormone treatment (Supplementary Fig. S2).

For quantification of GUS enzyme activity, four pericarp wall discs per fruit (two from each side of the pericarp wall mid-region) were taken using a cork borer (6 mm diameter), immediately frozen in liquid nitrogen and stored at –80 °C. Tissues from two fruits were pooled as a biological replicate, and three biological replicates were assessed per treatment. GUS enzyme activity was determined using a fluorescent assay protocol employing the GUS substrate 4-methylumbelliferyl glucuronide (MUG) adapted from Jefferson (1987; Supplementary Protocol S4).

### Hormonal analysis

Frozen tissues were lyophilized and ground to a fine powder. Each biological replicate consisted of tissues from a minimum of 10 fruits. Hormonal analysis was done by HPLC-tandem mass spectrometry at the National Research Council, Saskatoon, Canada (see Supplementary Protocol S5). We found that this method for extraction and quantification of auxins was sensitive for low levels of IAA and IAA-conjugates; however, it was not sensitive enough for quantification of low levels of 4-Cl-IAA (which occur in pea pericarp tissues; Magnus *et al.*, 1997).

### Statistical analyses

The data means in all experiments are the average of biological replicates (independent samples), and the number of biological replicates is given for each experiment within the specific data set. For the Arabidopsis root elongation and gene expression experiments, statistical significance was determined by performing a one-way ANOVA followed by a Holm–Sidak *post hoc* mean separation test (SigmaPlot 13; Systat Software Inc.). Two-tailed Student’s *t*-tests assuming unequal variance (Analysis ToolPak, Microsoft Excel 2013) were used for the pairwise comparisons of auxin-receptor gene expression in pollinated versus non-pollinated fruit tissues.

## Results and discussion

### TIR1/AFB family of auxin receptors in pea

Five genes belonging to the TIR1/AFB family of auxin receptors were identified in pea by screening a small-scale pea transcriptomic database. Two of these genes (*PsAFB2* and *PsAFB6,* GenBank accession numbers KY829120 and KY829119, respectively) have already been sequenced and reported in a US patent (Ozga *et al.*, 2010), one (*PsAFB4*, GenBank KX954126) was previously reported and characterized by Ligerot *et al.* (2017), and two were cloned and the sequences verified for the first time in this work (*PsTIR1a* and *PsTIRb*, GenBank KX954124 and KX954125, respectively). These genes represent four phylogenetic clades: TIR1, AFB2, AFB4, and AFB6 (Supplementary Fig. S3). Based on a transcriptome database screening, Ligerot *et al.* (2017) also reported five TIR1/AFB family members in pea. We further screened pea transcriptome sequences derived from diverse types of vegetative and reproductive tissues at different developmental stages (Franssen *et al.*, 2011; Kaur *et al.*, 2012; Duarte *et al.*, 2014; Sudheesh *et al.*, 2015) and found no additional putative pea auxin-receptor homologs, suggesting that the pea TIR1/AFB family is limited to only five members.

The leucine-rich repeat domain in the Arabidopsis TIR1 F-box protein contains a pocket for auxin binding. The bottom of this pocket contains an inositol hexakisphosphate (InsP_6_) molecule that probably acts as a structural co-factor. When auxin is bound in the TIR1 pocket, an Aux/IAA co-receptor protein fits into the pocket on top of the auxin molecule (Tan *et al.*, 2007). Comparison of Arabidopsis and pea auxin-receptor proteins shows that all the amino acids important for auxin binding and interaction with InsP_6_ in AtTIR1 (Tan *et al.*, 2007) are also conserved in the pea PsTIR1a, PsTIR1b, and PsAFB2 proteins, except for a single amino acid substitution, namely the V463 position in AtTIR1 represented by I457 in PsAFB2. A similar substitution was also seen in AtAFB2 (see amino acid alignment position 516 in Supplementary Fig. S4). The majority of AtTIR1 amino acids involved in the interactions with the Aux/IAA co-receptor were also conserved in the pea PsTIR1a, PsTIR1b, and PsAFB2 proteins (Supplementary Fig. S4).

Additionally, for the TIR1/AFB1 clade, a glycine (E) to lysine (K) change in the F-box domain of AtAFB1 compared to AtTIR1 reduces the ability of AtAFB1 to form a SKP1–CULLIN–F-box (SCF) ubiquitin ligase complex, which stabilizes the F-box protein and causes auxin resistance and associated growth defects, probably by protecting Aux/IAAs from degradation (Yu *et al.*, 2015). The E-to-K substitution in the F-box domain is shared by AFB1 homologs from members of the Brassicaceae and Cleomaceae, but not in homologs from the outgroup species papaya (*Carica papaya*) and cacao tree (*Theobroma cacao*; Yu *et al.*, 2015). Given this evolutionary grouping, the authors speculated that AFB1 and its orthologues retained a unique function after Brassicaceae diverged from the Capparaceae ~70 million years ago. Both PsTIR1 alleles possessed an E at this F-box domain position that is important for tethering TIR1 to SCF (Supplementary Fig. S5). However, the key E-to-K substitution was found in the F-box domain of the TIR1b allele of the *Trifolium* species in the subgenera Trifolium (*Trifolium pratense* and Trichocephalum (*T. subterraneum*), that as part of the Trifolieae tribe diverged from the Fabeae tribe in the Meso-Papilionoideae clade of the Fabaceae family ~16–23 million years ago (Ellison *et al.*, 2006; Schaefer *et al.*, 2012). Future detailed genetic studies in these or related legume species are required to determine whether the E-to-K change in the TIR1b orthologues in *Trifolium* imparts a unique function after the Trifolieae and Fabeae tribes diverged from their crown clade of Vicioid.

### Pea auxin receptors functionally interact with 2,4-D, IAA, and 4-Cl-IAA for auxin response

To determine whether *PsTIR1a* and *PsTIR1b* can complement *AtTIR1* in root elongation assays, they were separately introduced into the Arabidopsis *tir1-10* and *Attir1-10 afb2-3* mutants under the regulation of the Arabidopsis *TIR1* promoter. *Attir1-10* has a T-DNA insertion near the 5′-end region of the CDS and therefore is probably a null mutation (Parry *et al.*, 2009). In root growth assays, *Attir1-10* shows 2,4-D-insensitive phenotypes similar to that of *Attir1-1*. In *Atafb2-3*, a T-DNA insertion in the promoter region reduces *AtAFB2* expression (Parry *et al.*, 2009). As a transgenic control, *pAtTIR1::AtTIR1* was introduced into *Attir1-10* and *Attir1-10 afb2-3*.

In root growth assays, Arabidopsis *tir1-10* showed reduced auxin sensitivity (greater root elongation) in the presence of 2,4-D (50 nM and 70 nM) compared to that of the wild-type Col-0 seedlings (Supplementary Fig. S6). Compared to *Attir1-10*, the double-mutant *Attir1-10 afb2-3* showed a further reduction in 2,4-D sensitivity, which was similar to that shown for *Attir1-1 afb2-3* compared to *Attir1-1* (Parry *et al.*, 2009).

**Fig. 1.**
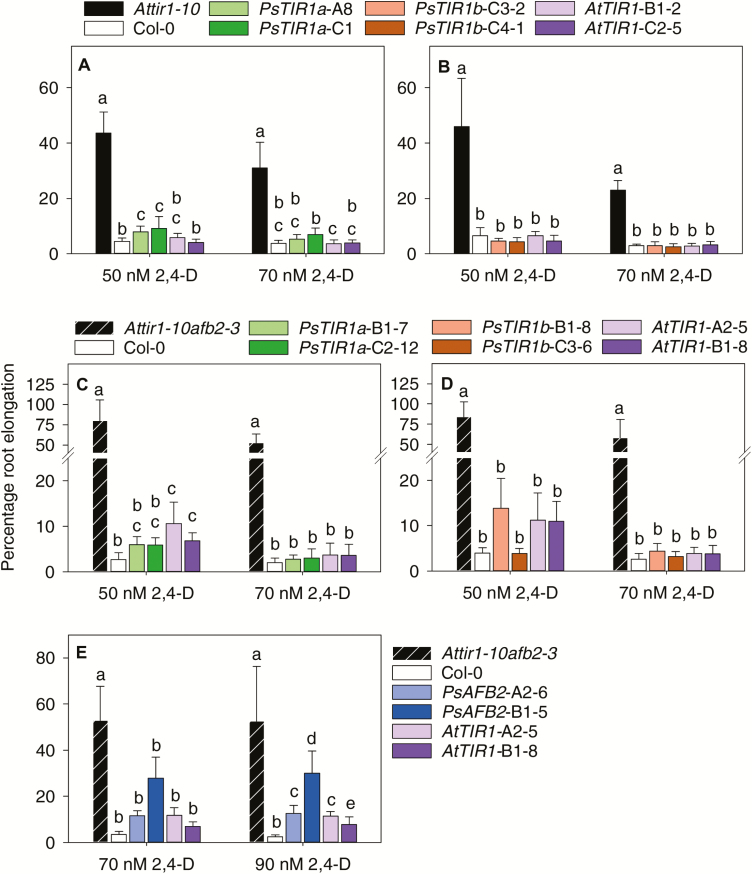
Functional characterization of pea auxin receptors with 2,4-D in Arabidopsis. Seedlings at 4 d old were transferred to media containing 2,4-D for 3 d. The seedlings assessed were Col-0, *Attir1-10*, *Attir1-10 afb2-3*, or those expressing (A, B) *pAtTIR1::cPsTIR1a* (*PsTIR1a*-A8 and C1), *pAtTIR1::cPsTIR1b* (*PsTIR1b*-C3-2 and C4-1) or *pAtTIR1::cAtTIR1* (*AtTIR1*-B1-2 and C2-5) in *Attir1-10*, or (C–E) *pAtTIR1::cPsTIR1a* (*PsTIR1a*-B1-7 and C2-12), *pAtTIR1::cPsTIR1b* (*PsTIR1b*-B1-8 and C3-6), *pAtTIR1::cPsAFB2* (*PsAFB2*-A2-6 and B1-5) or *pAtTIR1::cAtTIR1* (AtTIR1-A2-5 and B1-8) in *Attir1-10 afb2-3*. Root elongation is expressed as a percentage of the same genotype in medium without auxin. Data are means (±SD), *n*=8. Different letters indicate significant differences within the same auxin concentration as determined by one-way ANOVA and Holm–Sidak *post hoc* tests (*P*<0.05).

Transgenic Arabidopsis plants expressing *PsTIR1a* or *PsTIR1b* restored the 2,4-D inhibition of root growth in the Arabidopsis *tir1-10* and *tir1-10afb2-3* mutants to levels similar to those of mutants expressing the *AtTIR1* transgene, and this was consistent with the auxin root-inhibiting activity exhibited by the wild-type seedlings (Fig. 1A–D). The restoration of auxin-sensitive root growth in the Arabidopsis auxin-resistant mutants indicates that both *PsTIR1a* and *PsTIR1b* code for functional auxin receptors.

*PsAFB2* under the regulation of the *AtTIR1* promoter was introduced into Arabidopsis *Attir1-10 afb2-3*, as the AFB2 contribution to auxin response in the root was clearly shown in *Attir1-10 afb2-3*, but only a minor auxin sensitivity difference was observed in *Atafb2-3* (Parry *et al.*, 2009). The *Attir1-10 afb2-3* seedlings expressing *PsAFB2* exhibited reduced root length at 70 nM and 90 nM 2,4-D, indicating that *PsAFB2* also codes for a functional auxin receptor (Fig. 1E).

In Arabidopsis *tir1-1*, transgenic expression of *AtTIR1* under the *AtTIR1* promoter rescues the mutant’s root growth response to 2,4-D to the level of wild-type seedlings; however, *AtAFB1* driven by the *AtTIR1* promoter does not (Parry *et al.*, 2009). In contrast, both *PsTIR1a* and *PsTIR1b* were able to rescue the *tir1-10* mutant root growth phenotype when regulated by the *AtTIR1* promoter, and they both displayed a wild-type level of 2,4-D response at 70 nM (compare Col-0 to *PsTIR1a* and *PsTIR1b* transgenics; Fig. 1A, B). This difference in auxin response between AtTIR1/AFB1 and PsTIR1a/TIR1b may at least partially reside in an E-to-K substitution found in the F-box domain of AtAFB1 (reducing TIR1 tethering to SCF), but is not present in either PsTIR1a or PsTIR1b, as described above.

**Fig. 2.**
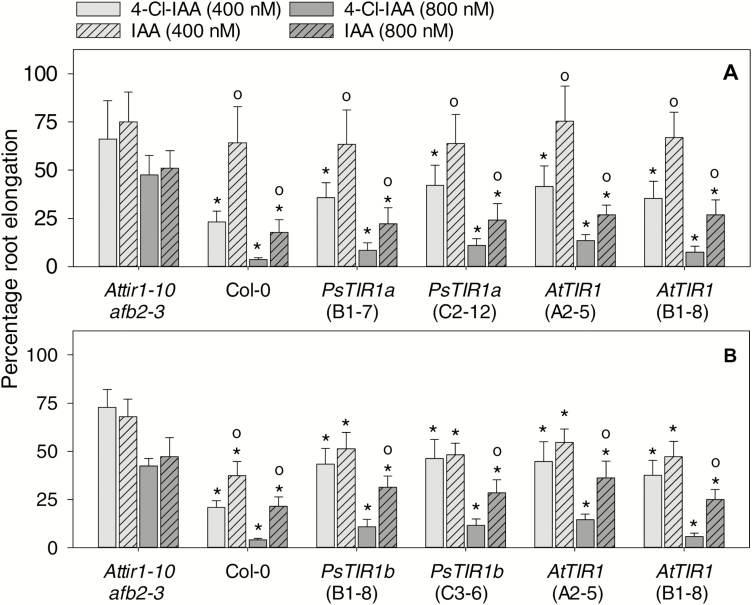
Functional characterization of pea auxin receptors with IAA and 4-Cl-IAA in Arabidopsis. Seedlings at 4 d old were transferred to media containing 0, 400, or 800 nM IAA or 4-Cl-IAA and grown for 3 d. Seedlings assessed were Col-0, *Attir1-10 afb2-3* or those expressing (A) *pAtTIR1::cPsTIR1a* (*PsTIR1a*-B1-7 and C2-12), (B) *pAtTIR1::cPsTIR1b* (*PsTIR1b*-B1-8 and C3-6), or (A, B) *pAtTIR1::cAtTIR1* (*AtTIR1*-A2-5 and B1-8). Root elongation in the auxin-containing medium is expressed as a percentage compared to the same genotype grown in medium without auxin. Data are means (±SD), *n*=8. Significant differences were determined by one-way ANOVA and Holm–Sidak *post hoc* tests (*P*<0.05): * means are significantly different from that of *Attir1-10 afb2-3* within the same auxin concentration; ° means from media containing IAA are significantly different from those containing 4-Cl-IAA within the same concentration and genotype.

Both 4-Cl-IAA and IAA were also effective in inhibiting Arabidopsis root elongation, but in the wild-type seedlings inhibition was greater with 4-Cl-IAA than IAA at the same auxin concentration (Fig. 2). In contrast, IAA inhibited root elongation to the same extent as 4-Cl-IAA in the *Attir1-10 afb2-3* mutant (Fig. 2). When *PsTIR1a*, *PsTIR1b*, or *AtTIR1* were expressed in *Attir1-10 afb2-3*, the transgenes restored the greater root inhibitory effect of 4-Cl-IAA, particularly at 800 nM, compared to that of IAA (Fig. 2).

It is possible that the chlorine molecule at the 4-position of the indole ring of 4-Cl-IAA (compared to the hydrogen moiety in IAA) modifies the hydrophobic interactions and van der Waals contacts in the auxin-binding pocket of TIR1 (as described by Tan *et al.*, 2007), leading to differences in auxin affinity to TIR1, in the binding of specific Aux/IAAs to the complex, and/or in the dissociation rate of the complexes. This is consistent with studies showing that the dissociation rate of the IAA7 degron motif DII and the TIR1 proteins differ depending on the type of auxin present (IAA, 1-NAA, picloram, or 2,4-D; Calderón Villalobos *et al.*, 2012). However, we cannot exclude the possibility that part of the differential response of IAA and 4-Cl-IAA is due to differences in conjugation, metabolism, or transport of each auxin. Overall, the absence of a differential auxin response to IAA and 4-Cl-IAA in the *tir1-10 afb2-3* mutant and the restoration of differential response in the mutants expressing *AtTIR1*, *PsTIR1a*, and *PsTIR1b* suggest that the stronger auxin response elicited by 4-Cl-IAA compared to IAA (at the same concentration) is at least partially mediated through TIR1.

### Developmental regulation of the expression of auxin-receptor genes in pea

#### Seedling tissues

**Fig. 3.**
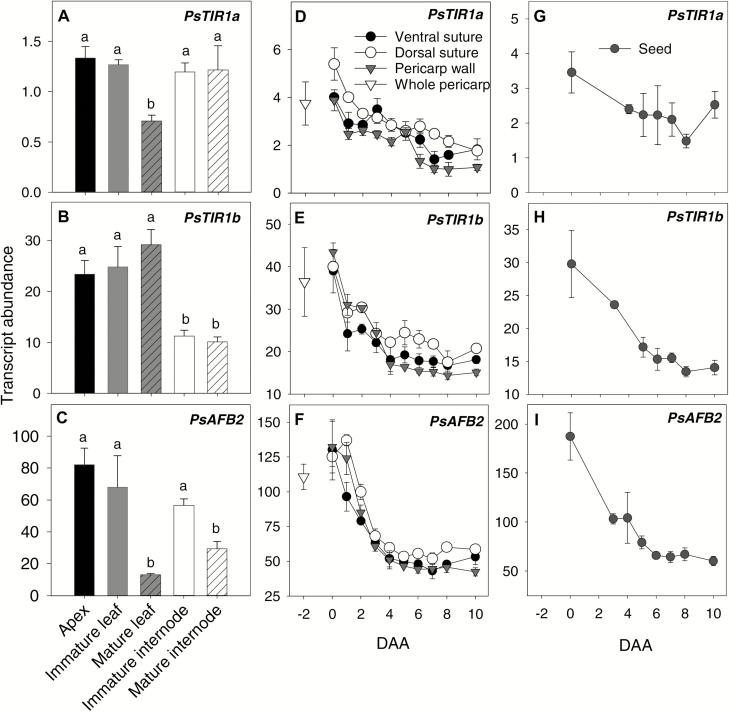
Developmental regulation of *PsTIR1a*, *PsTIR1b*, and *PsAFB2* transcript abundance in pea tissues. (A–C) Relative transcript abundance of auxin receptors in different tissues of 12-d-old seedlings. Data are means (±SD), *n*=3. Different letters indicate significant differences as determined by one-way ANOVA and Holm–Sidak *post hoc* tests (*P*<0.05). Relative transcript abundance of auxin receptors in (D–F) pericarps from pre-pollinated fruit at –2 d after anthesis (DAA) and in pericarp tissues (wall, dorsal vascular suture, and ventral vascular suture) from 0–10 DAA, and in (G–I) seeds from pollinated fruits from 0–10 DAA. Data are means (±SD), *n*=3 with the exception of dorsal suture at 0–3 DAA and seeds at 3 DAA and 4 DAA, where *n*=2. Each sample contained tissues from a minimum of three pericarps or from five seeds.

The *TIR1a*, *TIR1b*, and *PsAFB2* genes were expressed widely in the tissues but with varying transcript abundance patterns depending on the tissue type and developmental stage. In pea seedlings at 12 d after imbibition (DAI), *PsTIR1a* transcript abundance was similar across the vegetative tissues (shoot apexes, leaves, and internodes), except that levels decreased in leaves upon maturity (Fig. 3A). The pattern of tissue-specific transcript abundance of *PsTIR1b* differed from that of *PsTIR1a*, with transcript levels being lower in the internodes than in the shoot apex and leaf tissues, but no variation was observed between young and older tissues within a tissue type (Fig. 3B). The pattern of *PsAFB2* transcript abundance varied from that of *PsTIR1a* and *PsTIR1b*, with higher levels being observed in immature leaf and internode tissues than in the respective mature tissues (Fig. 3C).

#### Early pea fruit development

In pea, flowers self-pollinate approximately 24 h before flower opening (–1 DAA), and fertilization takes place by 0 DAA (Cooper, 1938). The developmental regulation of *PsTIR1a*, *PsTIR1b*, and *PsAFB2* transcript levels was analysed in –2 DAA pre-pollinated fruit, and in the pericarp wall, the dorsal and ventral pericarp vascular suture tissues, and the seeds of pollinated fruits from 0–10 DAA. In all pericarp tissues, *PsTIR1a*, *PsTIR1b*, and *PsAFB2* transcript levels were highest immediately after fertilization (0 DAA) and declined gradually with fruit development (Fig. 3D–F). In the developing seeds, the transcript abundance of *PsTIR1a* did not substantially change from 0–10 DAA. However, both *PsTIR1b* and *PsAFB2* gradually declined with development (by 2-fold and 5-fold from 0–10 DAA, respectively; Fig. 3G–I). Our data also suggested that *PsTIR1b* was more highly expressed (~10-fold) in pericarp tissues than *PsTIR1a* (Fig. 3D, E).

The presence of higher auxin-receptor transcript levels during early ovary growth followed by a gradual reduction with development appears to be a common trend in a number of species (*CsTIR1* and *CsAFB2* in cucumber, Cui *et al.*, 2014; *PslTIR1*, *PslAFB2*, and *PslAFB5* in plum, *Prunus salicina*, El-Sharkawy *et al.*, 2014; *SlTIR1* in tomato, Ren *et al.*, 2011). The higher abundance of auxin-receptor transcripts that is observed closely following fertilization events, in precocious fruit set, and in parthenocarpic fruit phenotypes in TIR1 overexpression lines of tomato (Ren *et al.*, 2011) supports the idea that an increased auxin sensitivity of the ovaries facilitates early ovary growth and development.

**Fig. 4.**
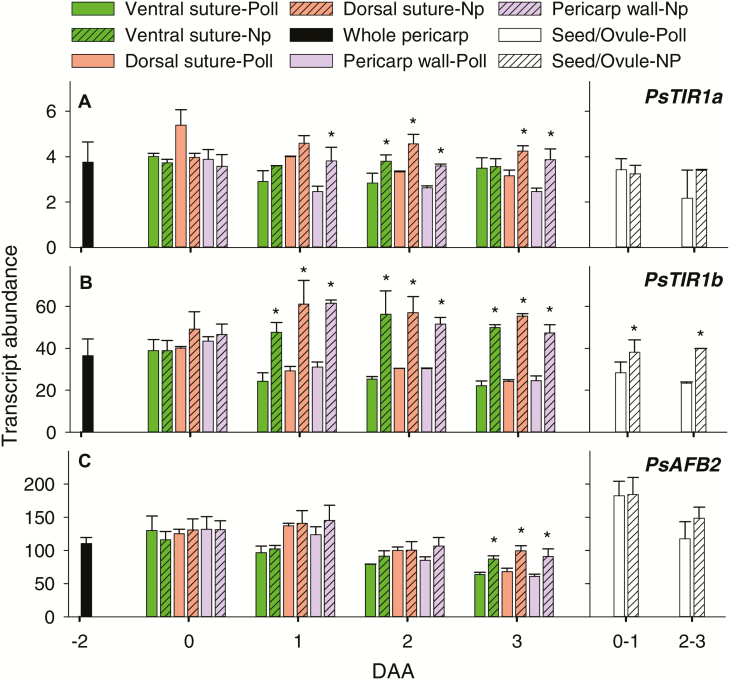
Relative transcript abundance of (A) *PsTIR1a*, (B) *PsTIR1b*, and (C) *PsAFB2* in different tissues of pollinated (Poll) and non-pollinated (Np) fruits of pea. Pre-pollinated pericarps are from fruits at –2 d after anthesis (DAA). Pericarp tissues (ventral vascular suture, dorsal vascular suture, and pericarp wall) are from pollinated or non-pollinated fruits at 0–3 DAA. Seeds (fertilized ovules from pollinated fruits) and non-fertilized ovules (from non-pollinated fruits) are from fruits at 0–1 DAA and 2–3 DAA. Data are means (±SD), *n*=3 for pre-pollinated pericarps and pericarp wall tissue; *n*=3 for pericarp ventral and dorsal sutures, except for a few samples where *n*=2 due to the limited size of tissue that was available. At 3 DAA, non-pollinated ovaries were still green and turgid. Pairwise mean comparisons were made using a two-tailed Student’s *t*-test (*P*<0.05): * means for non-pollinated fruits are significantly different from those for pollinated fruits within a tissue, gene, and DAA (*P*<0.05).

In pea, pericarp growth arrests by 1 DAA in the absence of pollination but pericarps remain green and turgid through to 3 DAA (Jayasinghege *et al.*, 2017). In non-pollinated pericarps, *PsTIR1b* transcript abundance was elevated ~2-fold by 1 DAA and remained elevated through to 3 DAA in all pericarp tissues compared to pollinated fruits (Fig. 4). *PsTIR1b* transcript abundance was also higher in non-fertilized ovules compared to seeds. *PsTIR1a* and *PsAFB2* expression in fruit/seed tissues was less tightly or not controlled by pollination events compared to that of *PsTIR1b*; however, by 3 DAA the transcript abundance of all three auxin receptors was higher in pericarp wall and dorsal suture tissues from non-pollinated compared to pollinated fruits (Fig. 4). Similarly, in the non-parthenocarpic cucumber cultivar 8419s-1, the transcript levels of *CsTIR1* and *CsAFB2* have been shown to increase in non-pollinated fruits but to decline in pollinated fruits during early fruit development (Cui *et al.*, 2014).

It is possible that increased expression of specific auxin-receptor genes is a response to low auxin levels or to signaling events within the pericarp tissue (due to the absence of developing seeds) that act to modify the auxin response in non-pollinated fruits. When Arabidopsis auxin receptors were studied using a synthetic system in yeast, the rates of IAA-induced Aux/IAA degradation were markedly different with TIR1 and AFB2 (Havens *et al.*, 2012). These data support the hypothesis that the pool of specific TIR1/AFB receptors in a cell or tissues at any one time may dictate specific auxin signaling outputs, leading to specific auxin responses. Changing the gene expression of specific TIR1/AFBs within a tissue or cell is one possible mechanism to change the pool of specific TIR1/AFB receptors. The prominent up-regulation of *PsTIR1b* in non-pollinated pea pericarps has the potential to modify the pool of TIR1/AFB receptors and hence the auxin response in this tissue.

### Developmental regulation of endogenous auxin levels and auxin activity in pea fruit

Due to the auxin-dependent activation of the *DR5* promoter (Ulmasov *et al.*, 1995, 1997), the localized GUS enzyme activity of *DR5::GUS* plants is commonly used as a marker of auxin maxima in plants (in pea; DeMason and Polowick, 2009). However, *DR5::GUS* expression relies on the transduction of the auxin signal to the *DR5* promoter, which may be affected by changes in the auxin signaling pathway, including the type and abundance of auxin receptors (TIR1/AFBs and Aux/IAAs) and transcription factors (auxin response factors) present in the tissue. Therefore, DR5-driven GUS enzyme activity may not necessarily represent the localized auxin concentrations, but it is an indication of localized auxin response (Vernoux *et al.*, 2011; Brunoud *et al.*, 2012). Quantification of auxin levels in conjunction with *DR5::GUS* expression assays can provide a more comprehensive picture of how changes in auxin concentration are related to auxin action within the ovary.

**Fig. 5.**
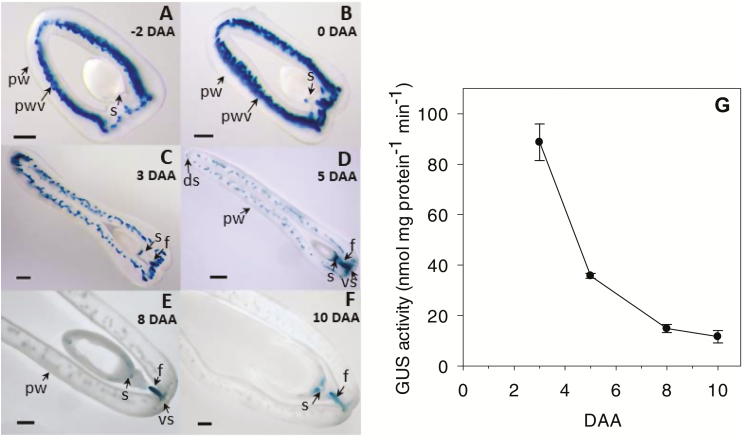
Developmental regulation of auxin activity in pea fruit. (A–F) Representative micrographs of GUS-stained fruit cross-sections and (G) GUS enzyme activity in the pericarp wall as detected by MUG assays in plants expressing the *GUS* gene under the regulation of the auxin-responsive *DR5* promoter (*DR5::GUS*). The micrographs show *DR5::GUS* expression in (A) pre-pollinated fruit at –2 d after anthesis (DAA) and (B–F) in pollinated fruit at 0, 3, 5, 8, and 10 DAA. ds, dorsal vascular suture; f, funiculus; pw, pericarp wall, pwv, pericarp wall vasculature; s, proximal end of the seed; vs, ventral vascular suture. Scale bars: (A, B) 500 µm and (C–F) 1000 µm. GUS enzyme activity was quantified in the central pericarp wall of pollinated fruits at 3–10 DAA. Data are means (±SD), *n*=3.

The IAA concentration in the pericarp of pollinated fruits was greatest at 0 DAA and decreased by ~2.5-fold by 3 DAA (on a fresh-weight basis, Table 1). In parallel with the decline in IAA, the concentration of the amide-conjugates IAA-aspartate (IAA-Asp) and IAA-glutamate (IAA-Glu) also declined from 0–3 DAA (Table 1). IAA-Asp and IAA-Glu are considered as irreversible IAA amide-conjugates destined for degradation (Ludwig-Müller, 2011), and IAA-Asp is the most abundant form of IAA amide-conjugate in pea (Sudi, 1964; Nordström and Eliasson, 1991). The reduction in the level of free IAA together with that of amide-conjugates suggests a reduction in pericarp IAA biosynthesis or a reduced IAA supply to the pericarp. Consistent with the reduction of IAA levels, the intense DR5-GUS staining observed in the pericarp vasculature of –2 DAA pre-pollinated and 0 DAA pollinated fruits also decreased by 3 DAA (Fig. 5A–C).

**Table 1.** IAA, IAA-aspartate (IAA-Asp), and IAA-glutamate (IAA-Glu) levels in pollinated ovaries from 0–8 d after anthesis (DAA), in non-pollinated ovaries (NP) at 3 DAA, and in seeds at 8 DAA of pea (cv I_3_, Alaska type; *DR5::GUS* plants) determined using HPLC-MS/MS and heavy-labelled internal standards

	**(ng g** ^**–1**^ **DW)**			**(ng g** ^**–1**^ **FW)**		
	**IAA**	**IAA-Asp**	**IAA-Glu**	**IAA**	**IAA-Asp**	**IAA-Glu**
Whole pericarp						
0 DAA	292±5	293±14	37±1	61±1	62±3	8±0
3 DAA	168±10	43±16	19±3	25±1	6±2	3±0
3 DAA-NP	38±13	<5	23±6	6±2	<1	4±1
5 DAA	136±1	209±62	13±4	18±0	27±8	2±1
8 DAA	135±11	124±47	8±0	23±2	21±8	1±0
Pericarp wall						
5 DAA	73±12	27±10	5±1	8±1	3±1	1±0
8 DAA	145±24	34±11	4±0	24±4	6±2	1±0
Ventral vascular suture						
5 DAA	774±10	1266±262	40±2	123±2	200±41	6±0
8 DAA	267±30	648±9	22±4	59±7	142±2	5±1
Dorsal vascular suture						
5 DAA	366	359	23	43	43	3
8 DAA	148±7	70±65	20±2	31±1	15±14	4±0
Seed						
8 DAA	32 262±700	84 497±6280	2704±52	5148±112	13 484±1002	431 ± 8

Data are means (±SD), *n*=2 with the exception of dorsal vascular suture at 5 DAA where only a single sample was analysed. Each replicate contained more than 50 pericarps at early developmental stages to a minimum of 10 pericarps at later developmental stages. Seeds were without the funiculus. IAA-alanine (IAA-Ala), IAA-leucine (IAA-Leu) and indole-3-butyric acid (IBA) were also analysed, but the levels were minute to non-detectable, with the exception of IAA-Ala in the seeds of 8 DAA fruits where the mean was 47.7±0.4 ng g^–1^ DW.

A further decrease in IAA levels (1.4-fold) in pericarps of pollinated fruits was observed from 3–5 DAA (Table 1). Similarly, Magnus *et al.* (1997) reported a 1.7-fold reduction in IAA levels from 3–6 DAA in pollinated pea pericarps (from 29 ng g^–1^ FW to 17 ng g^–1^ FW), whilst levels of 4-Cl-IAA in pollinated pericarps were lower than those of IAA and did not change from 3–6 DAA (~5 ng g^–1^ FW). The overall pericarp DR5-GUS staining intensity and enzyme activity also decreased from 3–5 DAA in the pericarps of pollinated fruits (Fig. 5C, D, G).

Young developing pea seeds contain high levels of auxin at 3 DAA and 6 DAA (ng g^–1^ FW, cv. Alaska I_3_; 3 DAA, IAA=393, 4-Cl-IAA=145; 6 DAA, IAA=1494, 4-Cl-IAA=231; Magnus *et al.*, 1997), and we also found high levels at 8 DAA (IAA=5148; Table 1). Localized GUS-staining auxin activity was observed towards the proximal end of the seed adjacent to the attachment to the funiculus (intense DR5-GUS staining at 3 DAA and 5 DAA; Fig. 5C, D), and in the funiculus (3–10 DAA; Fig. 5C–F) during early fruit development. Higher IAA and IAA amide-conjugate levels (Table 1) and GUS staining intensity (Fig. 5D) were also observed in the ventral vascular suture (where the seeds are attached to the pericarp through the funiculus) compared to the dorsal vascular suture (IAA 3-fold and IAA-Asp 5-fold higher on a fresh-weight basis), or to the pericarp wall (IAA 15-fold and IAA-Asp 67-fold higher on a fresh-weight basis) at 5 DAA.

Auxin-sensitive promoter-gene marker assays in Arabidopsis support the concept that fertilization-stimulated endosperm initiation is coupled to the production of auxin in the central cell, followed by accumulation of auxin in the seed coat (Figueiredo *et al.*, 2015, 2016) and the funiculus tissues (Robert *et al.*, 2018). Furthermore, in the absence of developing seeds within the pea ovary, IAA levels were ~4-fold lower and IAA-Asp was minimally detectable in the pericarps (compare 3 DAA pericarps from pollinated and non-pollinated fruits in Table 1). These data support the hypothesis that following fertilization, developing seeds produce auxin that can be transported to the pericarp, as suggested by the auxin concentration and *DR5::GUS* staining gradients (high to low, from the seeds and adjacent vascular tissues to the other pericarp tissues). Within the pea pericarp, auxin stimulates GA biosynthesis and other auxin-regulated processes to promote pericarp growth (Magnus *et al.*, 1997; Ozga *et al.*, 2009). The higher transcript levels of *PsTIR1b* (and to a lesser extent *PsTIRa* and *PsAFB2*) in pericarps from non-pollinated compared to pollinated fruits and in non-fertilized ovules compared to seeds (Fig. 4) indicate that the expression of auxin receptors (particularly *PsTIR1b*) may be regulated to modify the auxin response under low auxin conditions in these tissues.

From 5 DAA to 8 DAA, the free IAA and IAA-Asp levels decreased ~1.5-fold in the pericarp dorsal vascular suture and 2-fold in the ventral vascular suture (Table 1). In contrast to the pericarp vascular sutures, levels of free IAA and IAA-Asp increased from 5 DAA to 8 DAA in the pericarp wall tissues (by ~3-fold and ~2-fold, respectively). By 8 DAA, the pericarp has reached most of its maximal length and width, but the diameter rapidly increases from 6–12 DAA to accommodate the developing seeds (Ozga and Reinecke, 2003). The reduction in IAA level in the vascular suture tissues and the increased level in the pericarp wall from 5–8 DAA may indicate that auxin (IAA and 4-Cl-IAA) is transported from the seed through the vascular suture to the pericarp wall, and the higher auxin concentration in the wall facilitates rapid expansion of the diameter of the pericarp. Taken together, these data support the hypothesis that auxin concentration and response (through the regulation of auxin-receptor type and abundance) are developmentally and seed-regulated to coordinate seed and ovary growth.

### 4-Cl-IAA and IAA differentially modulate PsTIR1 transcript abundance and auxin activity in pea fruit

**Fig. 6.**
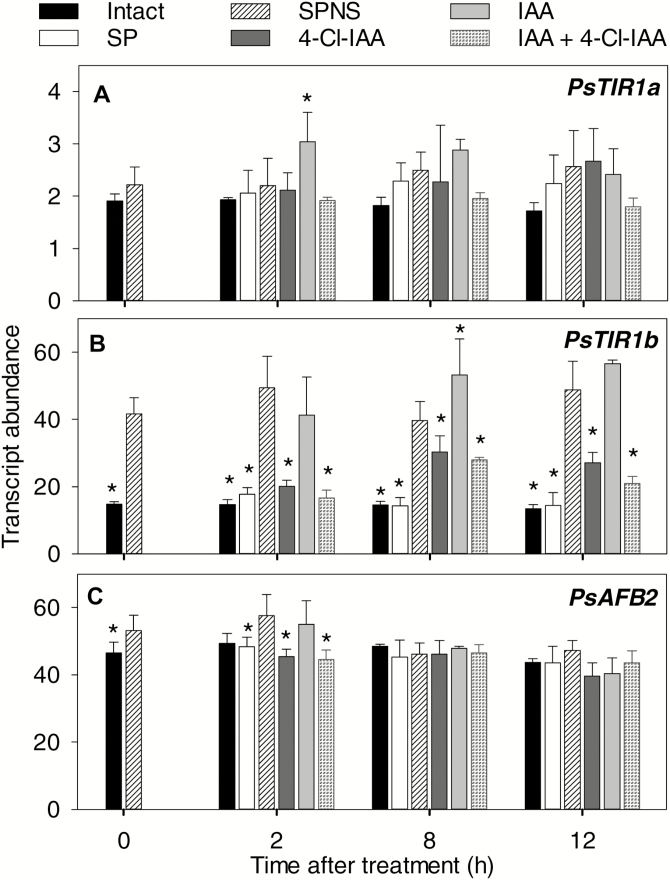
Effects of removal of pea seeds and auxin treatment on the relative transcript abundance of the pericarp auxin-receptor genes (A) *PsTIR1a*, (B) *PsTIR1b*, and (C) *PsAFB2*. At 2 d after anthesis, pollinated fruits were either left intact, split (split pericarps with seeds, SP), or split and deseeded (split pericarp no seeds, SPNS). Twelve hours after these treatments, the pericarps were treated with 4-Cl-IAA, IAA, or 4-Cl-IAA plus IAA (labelled as 0 h after treatment on the graphs). Data are means (±SD), *n*=3–8. * Means significantly different from that of the SPNS controls within time after treatment as determined by one-way ANOVA and Holm–Sidak post hoc tests (*P*≤0.05).

When seeds were present (intact and split pericarps, SP), transcript levels of *PsTIR1a*, *PsTIR1b*, and *PsAFB2* in the pericarps at 2 DAA remained relatively constant over the course of a 12-h assay (Fig. 6, Supplementary Fig. S7A–C). Seed removal (split pericarp no seeds, SPNS) increased the transcript abundance of *PsTIR1b* in the pericarp by ~3-fold but had a minimal or no effect on *PsTIR1a* and *PsAFB2*. Increased *PsTIR1b* transcript abundance in deseeded pericarps together with increased abundance in non-pollinated pericarps is consistent with the hypothesis that *PsTIR1b* expression is regulated in order to modify auxin signaling and response under low auxin conditions, and potentially in response to other seed-derived signals.

To assess auxins as a signal(s) regulating pericarp auxin-receptor expression, receptor abundance was determined in deseeded pericarps treated with IAA (which does not stimulate deseeded pericarp growth), 4-Cl-IAA (stimulates deseeded pericarp growth), or IAA plus 4-Cl-IAA. *PsTIR1a* and *PsAFB2* transcript abundance remained relatively constant in pericarps treated with IAA, 4-Cl-IAA, or both auxins (Fig. 6). In contrast, 4-Cl-IAA reduced the *PsTIR1b* transcript abundance (4-Cl-IAA or IAA plus 4-Cl-IAA compared to SPNS controls), but IAA had either a minimal or no effect. The increased abundance of *PsTIR1b* transcripts under low pericarp growth conditions (in the absence of developing seeds) and the suppression of transcript accumulation only in response to the growth-active auxin 4-Cl-IAA indicate that 4-Cl-IAA is a potential seed signal that regulates *PsTIR1b* expression.

**Fig. 7.**
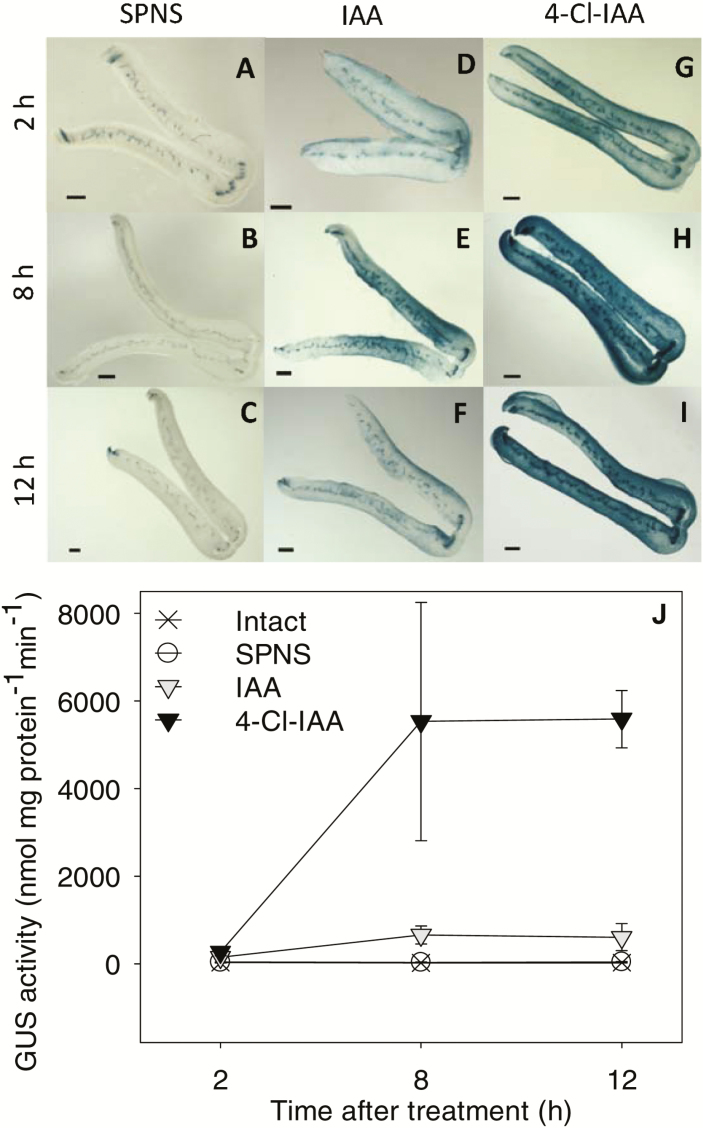
Effect of IAA and 4-Cl-IAA on *DR5::GUS* activity in pea fruits. (A–I) Representative micrographs of GUS-stained fruit cross-sections and (J) GUS enzyme activity as detected by MUG assays. Fruits at 2 d after anthesis from plants expressing *DR5::GUS* were split and deseeded (split pericarp no seeds, SPNS). Twelve h after this treatment, pericarps were treated with (A–C) aqueous 0.1% Tween 80 (SPNS, controls), (D–F) IAA, or (G–I) 4-Cl-IAA. Samples were harvested at 2 h, 8 h, or 12 h after hormone application. Scale bars are 500 µm. GUS enzyme activities at the 2-h time point were as follows: intact, 26.6±3.4; SPNS, 40.1±6.4; IAA, 153.0±21.8; 4-Cl-IAA, 267.2±89.6. Data are means (±SD), *n*=3.

Auxin application to the pericarp increased the mesocarp GUS staining intensity and GUS enzyme activity by 2 h after treatment (Fig. 7). The increased GUS responses confirm that both IAA and 4-Cl-IAA diffuse from the endocarp application site into the mesocarp tissue and initiate auxin responses within 2 h. By 12 h after auxin treatment, GUS enzyme activity was 9-fold higher in pericarps treated with 4-Cl-IAA than in those treated with IAA (Fig. 7J). The ability of 4-Cl-IAA to stimulate a stronger auxin response (GUS enzyme activity) than IAA at the same concentration in pea pericarps was associated with its ability to enrich the auxin-receptor transcript pool with *PsTIR1a* and *PsAFB2* transcripts (by decreasing *PsTIR1b* transcript abundance). It is possible that PsTIR1a and PsTIR1b have different binding affinities for 4-Cl-IAA and/or specific Aux/IAAs that vary the auxin response. Indeed, PsTIR1a and PsTIR1b have different amino acids at (respective) alignment positions 401 and 460 in their protein sequences (Supplementary Fig. S4), which have been identified as Aux/IAA (IAA7-)contacting amino acid residues of AtTIR1 (Tan *et al.*, 2007). The differential effects of 4-Cl-IAA and IAA on *DR5::GUS* expression and GUS enzyme activity may also be due in part to other factors including differential catabolism of these auxins in the pericarp tissue.

In the absence of pollination (or due to seed removal), ethylene evolution increases in the pericarp (Orzáez *et al.*, 1999; Johnstone *et al.*, 2005; Savada *et al.*, 2017). Application of 4-Cl-IAA but not IAA to deseeded pericarps inhibits the action of ethylene (Johnstone *et al.*, 2005). Therefore, to determine whether the regulation of auxin-receptor transcript abundance by IAA and 4-Cl-IAA was modulated through ethylene, deseeded pericarps were treated with either ethephon or STS alone or in the presence of IAA or 4-Cl-IAA. Neither the ethephon nor STS treatments had a clear effect on the transcript levels of *PsTIR1a*, *PsTIR1b*, or *PsAFB2*, indicating that their expression was not directly regulated by ethylene in this tissue (Supplementary Fig. S7 D–F).

Finally, to determine whether the differential regulation of auxin-receptor abundance by the two auxins was tissue dependent, we studied their transcript abundance in seedlings at 4 DAI exposed to IAA and 4-Cl-IAA. In contrast to pea pericarps where 4-Cl-IAA is stimulatory to growth and IAA is not (Reinecke *et al.*, 1995), both auxins inhibited seedling root elongation (Supplementary Fig. S8A). However, neither IAA nor 4-Cl-IAA clearly affected the transcript abundance of *PsTIR1a*, *PsTIR1b*, or *PsAFB2* in the plumule, epicotyl, or root-tip tissues of the seedlings (Supplementary Fig.S8B–D), suggesting that the modulation of auxin-receptor abundance by the two auxins is tissue specific.

### Conclusions

In summary, the ability of the *PsTIR1a*, *PsTIR1b*, and *PsAFB2* genes to complement the *Attir1-10 afb2-3* mutant and to restore auxin-sensitive root growth in Arabidopsis indicates that these genes code for functional auxin receptors. The auxin-receptor proteins coded by *PsTIR1a* and *PsTIR1b* elicited an auxin response in the presence of IAA or 4-Cl-IAA in Arabidopsis root growth assays, with 4-Cl-IAA having a stronger auxin response than IAA at the same concentration (800 nM). Furthermore, the loss of a stronger auxin response elicited by 4-Cl-IAA compared to that of IAA in the absence of TIR1 (in *Attir1-10 afb2-3*) and the regaining of this effect upon reintroduction of TIR1 (*AtTIR1*, *PsTIR1a*, or *PsTIR1b*) into *Attir1-10 afb2-3* suggest that at least part of this differential auxin response in Arabidopsis root growth assays is mediated through TIR1.

**Fig. 8.**
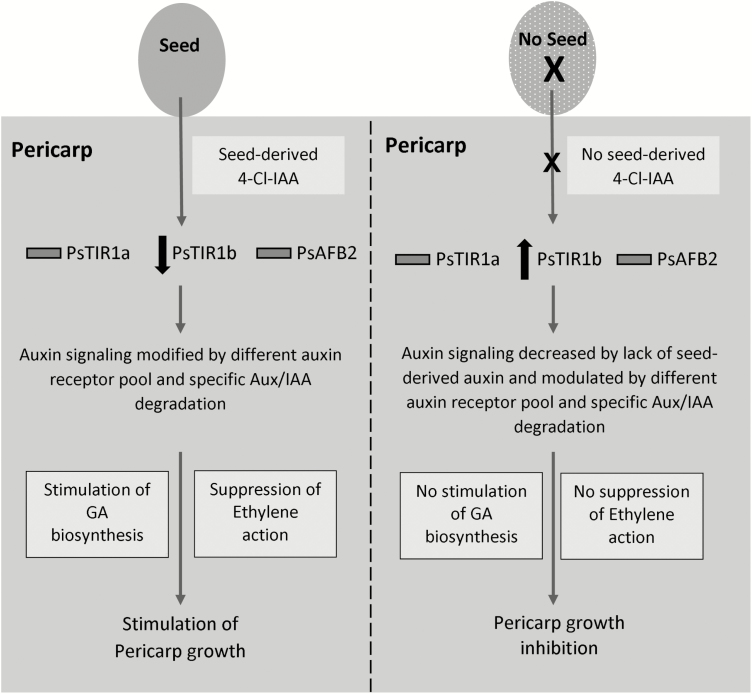
A working model for seed and auxin (4-Cl-IAA) stimulation of pea fruit growth. 4-Cl-IAA in the seed is transported to the pericarp, which leads to modulation of the pericarp auxin-receptor pool (through a decrease in *PsTIR1b* transcript abundance) that targets the degradation of specific Aux/IAA proteins, resulting in auxin-related changes in gene expression that facilitate pericarp growth. These seed/4-Cl-IAA-induced changes include the stimulation of GA biosynthesis and the suppression of ethylene action in the pericarp. In the absence of developing seeds, the lack of seed-derived auxin (4-Cl-IAA) modifies the make-up of the pericarp auxin-receptor pool (as noted by an increase in *PsTIR1b* transcript abundance) and reduces auxin signaling and activity, thus inhibiting pericarp growth.

Our data suggest that the markedly different effects of IAA and 4-Cl-IAA on pea fruit growth may at least partially involve differential modulation of the pericarp auxin-receptor population, and the substantial reduction of *PsTIR1b* transcript abundance induced by 4-Cl-IAA in the pericarp could suggest its importance in this tissue. We propose the following working hypothesis that seed and auxin (4-Cl-IAA) regulation of TIR1/AFBs (at least partially through the modification *PsTIR1b* transcript abundance) in the pea pericarp tissue affects the composition of the TIR1/AFB-Aux/IAA co-receptor complexes and their stabilities. This can lead to IAA- and 4-Cl-IAA-specific degradation of Aux/IAA proteins, thus initiating an auxin type-specific response in the pericarp (Fig. 8). The 4-Cl-IAA-specific response includes the stimulation of GA biosynthesis and inhibition of ethylene action (Ozga *et al.*, 2003, 2009; Johnstone *et al.*, 2005; Jayasinghege *et al.*, 2017) that facilitate pericarp growth. The lack of these 4-Cl-IAA-mediated pericarp responses (due to the absence of seeds) leads to pericarp senescence. The 4-Cl-IAA-dependent regulation of *PsTIR1b* transcript abundance in pea pericarps but not in seedling tissues supports a tissue-dependent modulation of auxin-receptor composition. The expression profiles of the Aux/IAA genes in different plant species also indicate tissue- and developmental stage-dependent modulation of Aux/IAA protein compositions (Kalluri *et al.*, 2007; Song *et al.*, 2009; Audran-Delalande *et al.*, 2012; Singh and Jain, 2015). Future analysis of the expression profiles of Aux/IAAs and the remaining TIR1/AFBs (PsAFB4 and PsAFB6) and evaluation of the IAA- and 4-Cl-IAA-dependent interactions of different co-receptor combinations will help to determine the specific co-receptor combinations that mediate auxin-dependent pea pericarp growth.

## Supplementary Data

Supplementary data are available at *JXB* online.

Fig. S1. Diagram of pea fruit tissues and confirmation of transgenic gene expression in Arabidopsis.

Fig. S2. Comparison of GUS staining in the pericarps from non-transgenic and *DR5::GUS*-expressing plants.

Fig. S3. A phylogenetic tree of predicted *P. sativum* auxin-receptor proteins.

Fig. S4. Sequence alignment of pea and Arabidopsis auxin-receptor proteins.

Fig. S5. Conservation of the TIR1 clade F-box protein domain in the Fabaceae subfamily Papilionoideae.

Fig. S6. Root elongation of Arabidopsis *tir1-10* and *tir1-10 afb2-3* mutant seedlings grown in the presence of 2,4-D.

Fig. S7. Effect of seed removal and ethylene on the relative transcript abundance of pea pericarp auxin receptors.

Fig. S8. Effect of IAA and 4-Cl-IAA on pea seedling growth and auxin-receptor abundance at 4 DAI.

Table S1. PCR primers used for gene cloning and verification.

Table S2. Primers and probes used for qRT-PCR.

Protocol S1. Pea TIR1/AFB family sequence analysis and construction of the phylogenetic tree.

Protocol S2. qRT-PCR assays.

Protocol S3. Creation of transgenic Arabidopsis plants and root growth analysis.

Protocol S4. GUS staining and quantification of GUS enzyme activity.

Protocol S5. Hormonal analysis.

Supplement Figures and Tables

## Authors contributions

CJ modified experimental designs for implementation and performed all experiments and data analysis except for those completed by HK and CN, and provided the first draft of the manuscript; JO conceived the original research, designed the experiments, interpreted the results, and was the primary editor of the manuscript; CN identified and sequenced *PsAFB2* and performed the first characterization of *PsAFB2* gene expression in pea fruit; HK performed qRT-PCR to verify the expression of the pea auxin receptors in transgenic Arabidopsis plants and edited the final manuscript; DR conceived the original research, created the *DR5::GUS* pea plants, identifying the pea *PsTIR1a* and *PsAFB4* homologs, provided technical assistance to CJ, and complemented the writing and editing of the manuscript.
