# Supplementary material for: TIR1 auxin receptors are implicated in the differential response to 4-Cl-IAA and IAA in developing pea fruit
Source: J Exp Bot. 2019 Jan 30;70(4):1239–53. doi: 10.1093/jxb/ery456 (PMC6382345; doi:10.1093/jxb/ery456)
Supplement: Supplement Figures and Tables [file ery456_suppl_supplementary-figures-s1-s2_tables-s1-s2.pdf]

SUPPLEMENTARY DATA

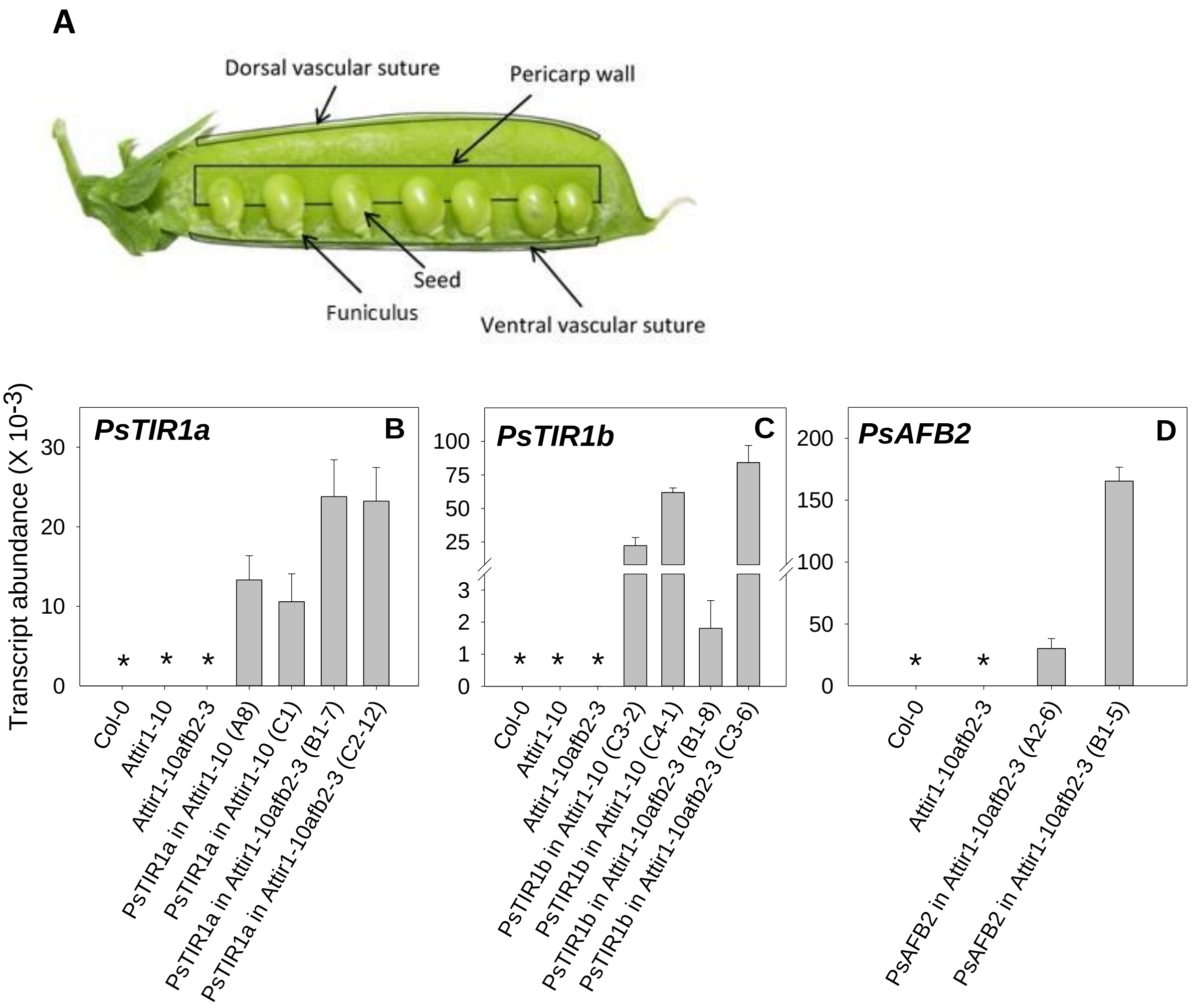

Supplemental Figure S1. Diagram of pea fruit tissues and confirmation of transgenic gene expression in Arabidopsis. (**A**) Pea fruit tissues from 0 to 10 days after anthesis (DAA) were dissected into seeds, pericarp wall, dorsal vascular suture, and ventral vascular suture tissues and used for transcript abundance analysis, and at 5 and 8 DAA for hormonal analysis. Representative fruit is approximately at 9 DAA. (**B**) Relative transcript abundance of pea auxin receptor genes *PsTIR1a*, *PsTIR1b* and *PsAFB2* in the leaves of approximately 1-month-old Arabidopsis transgenic and control lines. Data are means ± SD (n=3-4). Each sample is composed of leaves from about three to six plants. * No transgene mRNA transcripts detected.

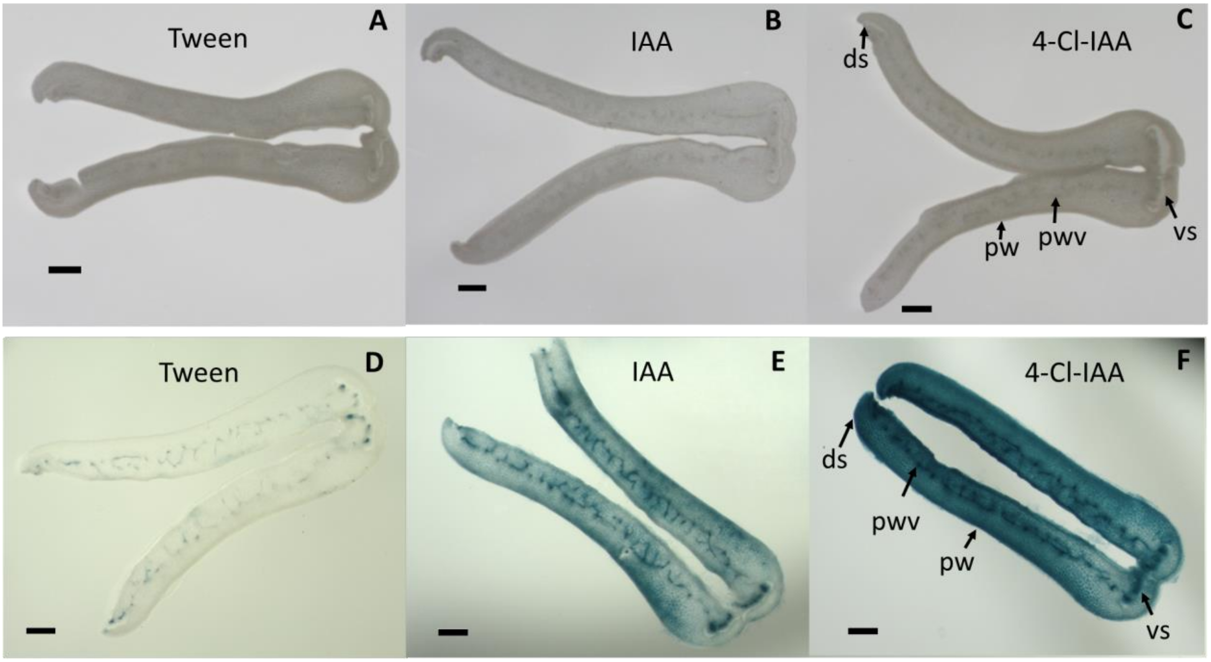

Supplemental Figure S2. Comparison of GUS staining in the pericarps from non-transgenic and *DR5::GUS* expressing plants. Two days after anthesis pollinated pericarps from non-transgenic (**A-C**) or transgenic plants expressing *DR5::GUS* (**D-F**) were split and deseeded (SPNS). Twelve hours after deseeding, pericarp were treated with 0.1% aqueous Tween 80 (**A** and **D**), IAA (50 μM; **B** and **E**) or 4-Cl-IAA (50 μM; **C** and **F**). Samples were collected for GUS staining 8 h after hormone treatment. Pericarp wall (pw), pericarp wall vasculature (pwv), dorsal vascular suture (ds), ventral vascular suture (vs). Scale bar = 500 μm.

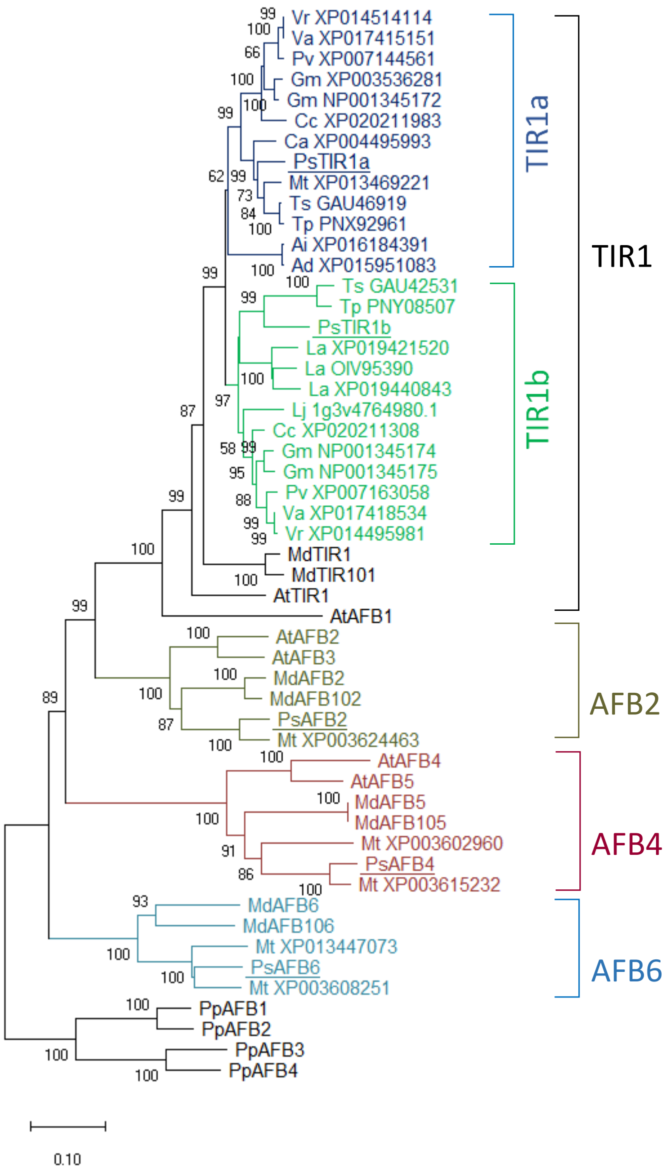

Supplemental Figure S3. A phylogenetic tree of predicted *P. sativum* auxin receptor proteins. A neighbor-joining tree was created with auxin receptor sequences of pea (PsTIR1a, PsTIR1b, PsAFB2, PsAFB4 and PsAFB6; GenBank accession numbers, KX954124, KX954125, KY829120, KX954126 and KY829119, respectively), *Arabidopsis thaliana* (At; *Parry et al.*, 2009), *Malus x domestica* (Md; Devoghalaere *et al.*, 2012) and *Medicago truncatula* (Mt). In addition, TIR1 orthologs of the legume species *Arachis duranensis* (Ad), *Arachis ipaensis* (Ai), *Cicer arietinum* (Ca), *Cajanus cajan* (Cc), *Glycine max* (Gm), *Lotus japonicus* (Lj), *Lupinus angustifolius* (La), *Phaseolus vulgaris* (Pv), *Trifolium pretense* (Tp), *Trifolium subterraneum* (Ts), *Vigna angularis* (Va) and *Vigna radiata* var. *radiata* (Vr) were also included. The tree was rooted with the auxin receptor sequences of the moss *Physcomitrella patens* (Pp; Parry *et al.*, 2009; Prigge *et al.*, 2010). Numbers in the branches represent the percentage bootstrap support. Bootstrap values with greater than 50 % branch support are shown.

Unconserved 0 1 2 3 4 5 6 7 8 9 10 Conserved

```
          ..........10 ..........20 ..........30 ..........40 ..........50
AtTIR1      ---------- ---------- ---------- ---------- --MQKRI---
PsTIR1a     ---------- ---------- ---------- ---------- --MQ-RV---
AtAFB1      ---------- ---------- ---------- ---------- ------M---
PsTIR1b     ---------- ---------- ---------- ---------- --MQ-KM---
PsAFB6      ---------- ---------- ---------- ---------- --MEPQTMNP
AtAFB2      ---------- ---------- ---------- ---------- ----------
AtAFB3      ---------- ---------- ---------- ---------- ----------
PsAFB2      ---------- ---------- ---------- ---------- ----------
AtAFB4      MTEEDSSAKM SEDVEKYLNL NPPCSSSSSS SSAATFTNKS RNFKSSPPPC
AtAFB5      MTQDRSEMSE DDDDQQSPPL DLPSTAIADP CSSSSSPNKS RNCISNSQTF
PsAFB4      MRENHPPTTT PDLLARGEIA ESSTSKNRTG SSE---PFPG SSLTENPSPF
Consistency 0000000000 0000000000 0000000000 0000000000 0021022000
```

```
          ..........60 ..........70 ..........80 ..........90 ..........100
AtTIR1      ALSFPEEVLE HVFSFIQLDK DRNSVSLVCK SWYEIERWCR RKVFIGNCYA
PsTIR1a     AYSFPEEVLE HVFSFIESDT DRGSISLVCK SWYEIERWCR RRVFVGNCYA
AtAFB1      GLRFPPKVLE HILSFIDSNE DRNSVSLVCK SWFETERKTR KRVFVGNCYA
PsTIR1b     TNRFPEEVLE YVFSFIQCDK DRNSISLVCK SWYEIERWCR RQIFVGNCYA
PsAFB6      SSVFPDEVLE RILSMVKSRK DKSSVSLVCK DWFDAERWSR KNVFIGNCYS
AtAFB2      MNYFPDEVIE HVFDFVTSHK DRNAISLVCK SWYKIERYSR QKVFIGNCYA
AtAFB3      MNYFPDEVIE HVFDFVASHK DRNSISLVCK SWHKIERFSR KEVFIGNCYA
PsAFB2      MNYFPDEVIE HVFDYVVSHS DRNSLSLVCK SWYRIEGFTR KRVFIGNCYS
AtAFB4      PDHVLENVLE NVLQFLTSRC DRNAVSLVCR SWYRVEAQTR LEVFIGNCYS
AtAFB5      PDHVLENVLE NVLQFLDSRC DRNAASLVCK SWWRVEALTR SEVFIGNCYA
PsAFB4      PDQVLENVLE NVLHFLSSRK DRNAASLVCR SWYRAEALTR SDLFIGNCYA
Consistency 3437676*8* 6965884745 *9887****8 8*766*635* 559*9****8
```

```
          ..........110 ..........120 ..........130 ..........140 ..........150
AtTIR1      VSPATVIRRF PKVRSVELKG KPHFADFNLV PDGWGGYVYP WIEAMSSSYT
PsTIR1a     VSPAMVIKRF PKVRSITLKG KPHFADFNLV PEGWGGYVCP WIKAMAASYP
AtAFB1      VSPAAVTRRF PEMRSLTLKG KPHFADYNLV PDGWGGYAWP WIEAMAAKSS
PsTIR1b     VSPVTVTKRF PELRSISLKG KPHFADFNLV PEGWGGFVSP WIAAMACGLP
PsAFB6      VTPEILTQRF PNVRSVTLKG KPRFSDFNLV PANWGADIHP WLVVFAEKYP
AtAFB2      INPERLLRRF PCLKSLTLKG KPHFADFNLV PHEWGGFVLP WIEALARSRV
AtAFB3      INPERLIRRF PCLKSLTLKG KPHFADFNLV PHEWGGFVHP WIEALARSRV
PsAFB2      ISPERLVERF PDFKSLTLKG KPHFADFSLV PHGWGGFVYP WIEALAKSRV
AtAFB4      LSPARLIHRF KRVRSLVLKG KPRFADFNLM PPNWGAQFSP WVAATAKAYP
AtAFB5      LSPARLTQRF KRVRSLVLKG KPRFADFNLM PPDWGANFAP WVSTMAQAYP
PsAFB4      LSPRRATARF SHIKSVTVKG KPRFADFDLM PVDWGAHFTP WVTSLAQAYP
Consistency 87*55765** 6378*869** **6*9*98*8 *35**7463* *957694645
```

```
          ..........160 ..........170 ..........180 ..........190 ..........200
AtTIR1      WLEEIRLKRM VVTDDCLELI AKSFKNFKVL VLSSCEGFST DGLAAIAATC
PsTIR1a     CLQEIRLKRM VITDDSLDLI AKSFKNFTVL VLTSCEGFTT EGLAAIAANC
AtAFB1      SLEEIRMKRM VVTDECLEKI AASFKDFKVL VLTSCEGFST DGIAAIAATC
PsTIR1b     LLEEIRLKRM VITDESLELI AKSFKNFKVL VLISCEGFTT EGLAAIASNC
PsAFB6      FLEELRLKRM VVTDESLEFL AFSFPNFKAL SLLSCDGFST DGLAAVATNC
AtAFB2      GLEELRLKRM VVTDESLELL SRSFVNFKSL VLVSCEGFTT DGLASIAANC
AtAFB3      GLEELRLKRM VVTDESLDLL SRSFANFKSL VLVSCEGFTT DGLASIAANC
PsAFB2      GLEELRLKRM VVSDESLELL SRSFMNFKSL VLVSCEGFTT DGLAAVAANC
AtAFB4      WLEKVHLKRM FVTDDDLALL AESFPGFKEL TLVCCEGFGT SGIAIVANKC
AtAFB5      CLEKVDLKRM FVTDDDLALL ADSFPGFKEL ILVCCEGFGT SGISIVANKC
PsAFB4      WLEKLHLKRM SVTDKDLGLI ADSFVGFREL LLACCEGFGT PGLAVIASKC
Consistency 2*98869*** 699*75*578 84**46*84* 7*66*9**5* 6*8969*66*
```

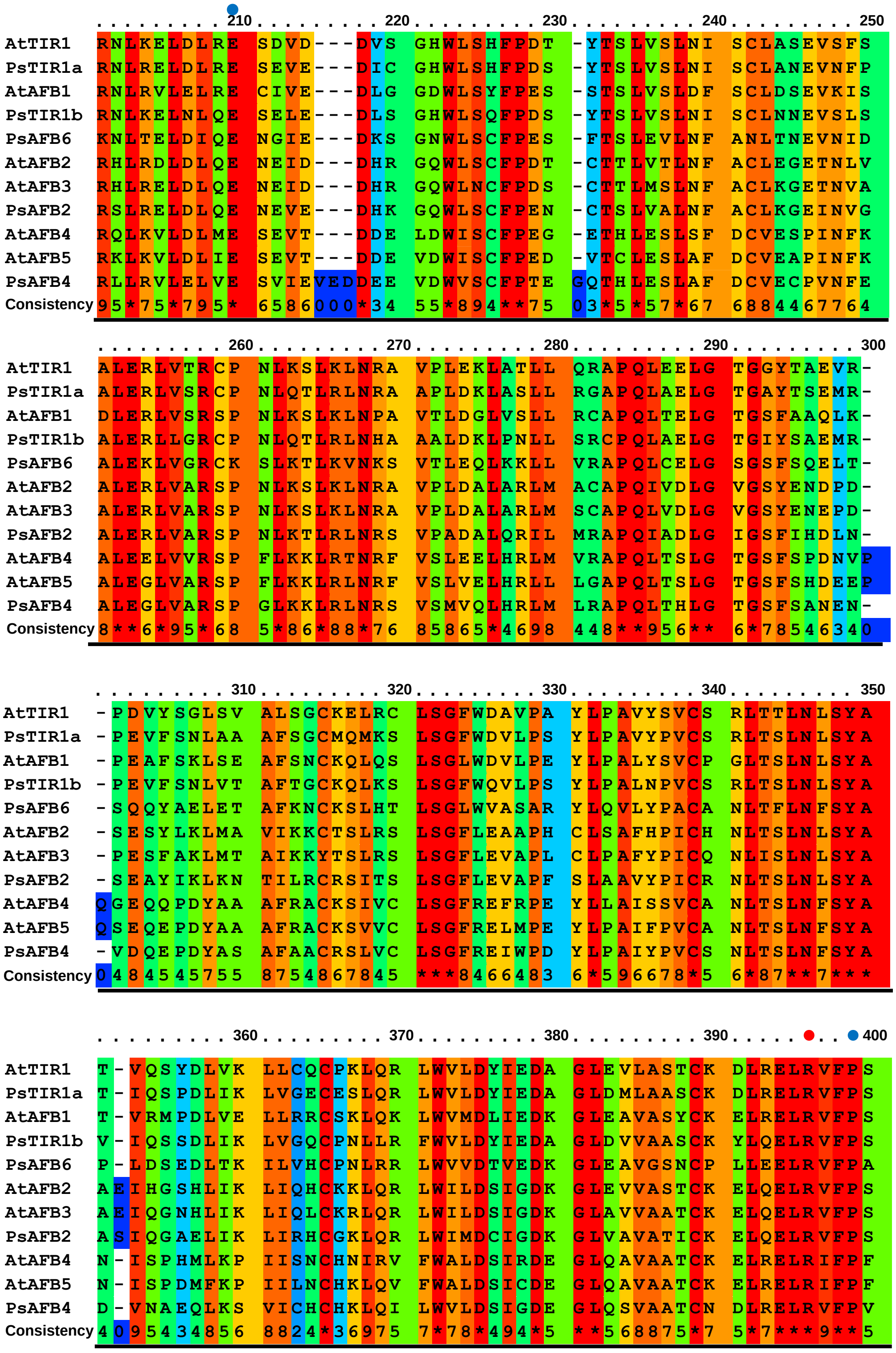
. . . . 210 . . . . 220 . . . . 230 . . . . 240 250
AtTIR1 RNLKELDLRE SDVD---DVS GHWLSHFPDT -YTSLVSLNI SCLASEVSFS
PsTIR1a RNLRELDLRE SEVE---DIC GHWLSHFPDS -YTSLVSLNI SCLANEVNFP
AtAFB1 RNLRVLELRE CIVE---DLG GDWLSYFPES -STSLVSLDF SCLDSEVKIS
PsTIR1b RNLKELNLQE SELE---DLS GHWLSQFPDS -YTSLVSLNI SCLNNEVSLS
PsAFB6 KNLTELDIQE NGIE---DKS GNWLSCFPES -FTSLEVLNF ANLTNEVNID
AtAFB2 RHLRDLDLQE NEID---DHR GQWLSCFPDT -CTTLVTLNF ACLEGETNLV
AtAFB3 RHLRELDLQE NEID---DHR GQWLNCFPDS -CTTLMSLNF ACLKGETNVA
PsAFB2 RSLRELDLQE NEVE---DHK GQWLSCFPEN -CTSLVALNF ACLKGEINVG
AtAFB4 RQLKVLDLME SEVT---DDE LDWISCFPEG -ETHLESLSF DCVESPINFK
AtAFB5 RKLKVLDLIE SEVT---DDE VDWISCFPED -VTCLESLAF DCVEAPINFK
PsAFB4 RLLRVLELVE SVIEVEDDEE VDWVSCFPTE GQTHLESLAF DCVECPVNFE
Consistency 95*75*795* 6586000*34 55*894**75 03*5*57*67 6884467764
. . . . 260 . . . . 270 . . . . 280 . . . . 290 300
AtTIR1 ALERLVTRCP NLKSLKLNRA VPLEKLATLL QRAPQLEELG TGGYTAEVR-
PsTIR1a ALERLVSRCP NLQTLRLNRA APLDKLASLL RGAPQLAELG TGAYTSEMR-
AtAFB1 DLERLVSRSP NLKSLKLNPA VTLDGLVSLL RCAPQLTELG TGSFAAQLK-
PsTIR1b ALERLLGRCP NLQTLRLNHA AALDKLPNLL SRCPQLAELG TGIYSAEMR-
PsAFB6 ALEKLVGRCK SLKTLKVNKS VTLEQLKKLL VRAPQLCELG SGSFSQELT-
AtAFB2 ALERLVARSP NLKSLKLNRA VPLDALARLM ACAPQIVDLG VGSYENDPD-
AtAFB3 ALERLVARSP NLKSLKLNRA VPLDALARLM SCAPQLVDLG VGSYENEPD-
PsAFB2 ALERLVARSP NLKTLRLNRS VPADALQRIL MRAPQIADLG IGSFIHDLN-
AtAFB4 ALEELVVRSP FLKKLRTNRF VSLEELHRLM VRAPQLTSLG TGSFSPDNVP
AtAFB5 ALEGLVARSP FLKKLRLNRF VSLVELHRLL LGAPQLTSLG TGSFSHDEEP
PsAFB4 ALEGLVARSP GLKKLRLNRS VSMVQLHRLM LRAPQLTHLG TGSFSANEN-
Consistency 8**6*95*68 5*86*88*76 85865*4698 448**956** 6*78546340
. . . . 310 . . . . 320 . . . . 330 . . . . 340 350
AtTIR1 -PDVYSGLSV ALSGCKELRC LSGFWDAVPA YLPAVYSVCS RLTTLNLSYA
PsTIR1a -PEVFSNLAA AFSGCMQMKS LSGFWDVLPS YLPAVYPVCS RLTSLNLSYA
AtAFB1 -PEAFSKLSE AFSNCKQLQS LSGLWDVLPE YLPALYSVCP GLTSLNLSYA
PsTIR1b -PEVFSNLVT AFTGCKQLKS LSGFWQVLPS YLPALNPVCS RLTSLNLSYA
PsAFB6 -SQQYAELET AFKNCKSLHT LSGLWVASAR YLQVLYPACA NLTFLNFSYA
AtAFB2 -SESYLKLMA VIKKCTSLRS LSGFLEAAPH CLSAFHPICH NLTSLNLSYA
AtAFB3 -PESFAKLMT AIKKYTSLRS LSGFLEVAPL CLPAFYPICQ NLISLNLSYA
PsAFB2 -SEAYIKLKN TILRCRSITS LSGFLEVAPF SLAAVYPICR NLTSLNLSYA
AtAFB4 QGEQQPDYAA AFRACKSIVC LSGFREFRPE YLLAISSVCA NLTSLNFSYA
AtAFB5 QSEQEPDYAA AFRACKSVVC LSGFRELMPE YLPAIFPVCA NLTSLNFSYA
PsAFB4 -VDQEPDYAS AFAACRSLVC LSGFREIWPD YLPAIYPVCS NLTSLNFSYA
Consistency 0484545755 8754867845 ***8466483 6*596678*5 6*87**7***
. . . . 360 . . . . 370 . . . . 380 . . . . 390 400
AtTIR1 T-VQSYDLVK LLCQCPKLQR LWVLDYIEDA GLEVLASTCK DLRELRVFPS
PsTIR1a T-IQSPDLIK LVGECESLQR LWVLDYIEDA GLDMLAASCK DLRELRVFPS
AtAFB1 T-VRMPDLVE LLRRCSKLQK LWVMDLIEDK GLEAVASYCK ELRELRVFPS
PsTIR1b V-IQSSDLIK LVGQCPNLLR FWVLDYIEDA GLDVVAASCK YLQELRVFPS
PsAFB6 P-LDSEDLTK ILVHCPNLRR LWVVDTVEDK GLEAVGSNCP LLEELRVFPA
AtAFB2 AEIHGSHLIK LIQHCKKLQR LWILDSIGDK GLEVVASTCK ELQELRVFPS
AtAFB3 AEIQGNHLIK LIQLCKRLQR LWILDSIGDK GLAVVAATCK ELQELRVFPS
PsAFB2 ASIQGAELIK LIRHCGKLQR LWIMDCIGDK GLVAVATICK ELQELRVFPS
AtAFB4 N-ISPHMLKP IISNCHNIRV FWALDSIRDE GLQAVAATCK ELRELRIFPF
AtAFB5 N-ISPDMFKP IILNCHKLQV FWALDSICDE GLQAVAATCK ELRELRIFPF
PsAFB4 D-VNAEQLKS VICHCHKLQI LWVLDSIGDE GLQSVAATCN DLRELRVFPV
Consistency 4095434856 8824*36975 7*78*494*5 **568875*7 5*7***9**5

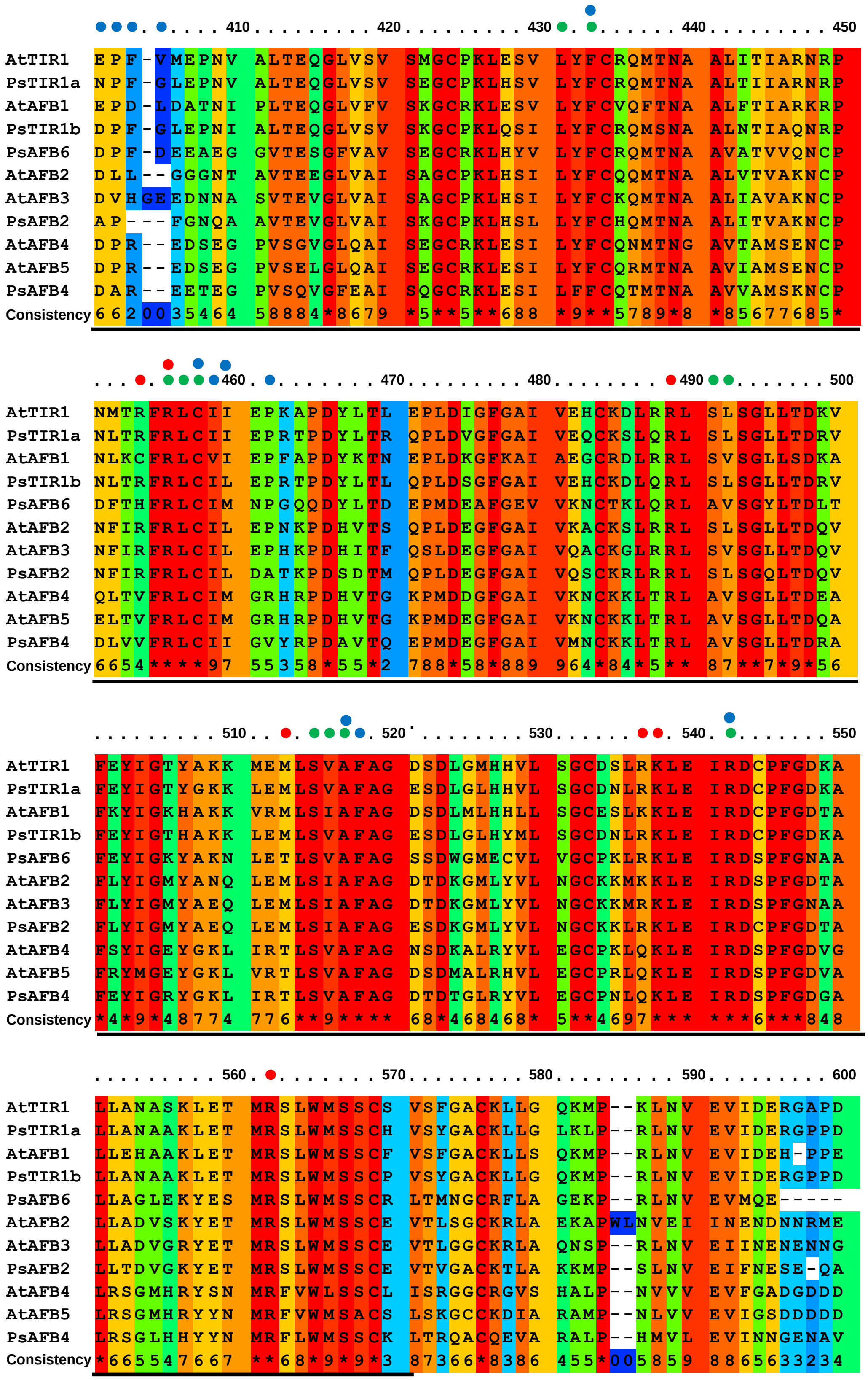
410 420 430 440 450
AtTIR1 EPF-VMEPNV ALTEQGLVSV SMGCPKLESV LYFCRQMTNA ALITIARNRP
PsTIR1a NPF-GLEPNV ALTEQGLVSV SEGCPKLHSV LYFCRQMTNA ALTIIARNRP
AtAFB1 EPD-LDATNI PLTEQGLVFV SKGCRKLESV LYFCVQFTNA ALFTIARKRP
PsTIR1b DPF-GLEPNI ALTEQGLVSV SKGCPKLQSI LYFCRQMSNA ALNTIAQNRP
PsAFB6 DPF-DEEAEG GVTESGFVAV SEGCRKLHYV LYFCRQMTNA AVATVVQNCP
AtAFB2 DLL--GGGNT AVTEEGLVAI SAGCPKLHSI LYFCQQMTNA ALVTVAKNCP
AtAFB3 DVHGEEDNNA SVTEVGLVAI SAGCPKLHSI LYFCKQMTNA ALIAVAKNCP
PsAFB2 AP---FGNQA AVTEVGLVAI SKGCPKLHSL LYFCHQMTNA ALITVAKNCP
AtAFB4 DPR--EDSEG PVSGVGLQAI SEGCRKLESI LYFCQNMTNG AVTAMSENCP
AtAFB5 DPR--EDSEG PVSELGLQAI SEGCRKLESI LYFCQRMTNA AVIAMSENCP
PsAFB4 DAR--EETEG PVSQVGFEAI SQGCRKLESI LFFCQTMTNA AVVAMSKNCP
Consistency 6620035464 58884*8679 *5**5**688 *9**5789*8 *85677685*
460 470 480 490 500
AtTIR1 NMTRFRLCII EPKAPDYLTL EPLDIGFGAI VEHCKDLRRL SLSGLLTDKV
PsTIR1a NLTRFRLCII EPRTPDYLTR QPLDVGFGAI VEQCKSLQRL SLSGLLTDRV
AtAFB1 NLKCFRLCVI EPFAPDYKTN EPLDKGFKAI AEGCRDLRRL SVSGLLSDKA
PsTIR1b NLTRFRLCIL EPRTPDYLTL QPLDSGFGAI VEHCKDLQRL SLSGLLTDRV
PsAFB6 DFTHFRLCIM NPGQQDYLTD EPMDEAFGEV VKNCTKLQRL AVSGYLTDLT
AtAFB2 NFIRFRLCIL EPNKPDHVTS QPLDEGFGAI VKACKSLRRL SLSGLLTDQV
AtAFB3 NFIRFRLCIL EPHKPDHITF QSLDEGFGAI VQACKGLRRL SVSGLLTDQV
PsAFB2 NFIRFRLCIL DATKPDSDTM QPLDEGFGAI VQSCKRLRRL SLSGQLTDQV
AtAFB4 QLTVFRLCIM GRHRPDHVTG KPMDDGFGAI VKNCKKLTRL AVSGLLTDEA
AtAFB5 ELTVFRLCIM GRHRPDHVTG KPMDEGFGAI VKNCKKLTRL AVSGLLTDQA
PsAFB4 DLVVFRLCII GVYRPDAVTQ EPMDEGFGAI VMNCKKLTRL AVSGLLTDRA
Consistency 6654****97 55358*55*2 788*58*889 964*84*5** 87**7*9*56
510 520 530 540 550
AtTIR1 FEYIGTYAKK MEMLSVAFAG DSDLGMHHVL SGCDSLRKLE IRDCPFGDKA
PsTIR1a FEYIGTYGKK LEMLSVAFAG ESDLGLHHVL SGCDNLRKLE IRDCPFGDKA
AtAFB1 FKYIGKHAKK VRMLSIAFAG DSDLMLHHLL SGCESLKKLE IRDCPFGDTA
PsTIR1b FEYIGTHAKK LEMLSVAFAG ESDLGLHYML SGCDNLRKLE IRDCPFGDKA
PsAFB6 FEYIGKYAKN LETLSVAFAG SSDWGMECVL VGCPKLRKLE IRDSPFGNAA
AtAFB2 FLYIGMYANQ LEMLSIAFAG DTDKGMLYVL NGCKKMKKLE IRDSPFGDTA
AtAFB3 FLYIGMYAEQ LEMLSIAFAG DTDKGMLYVL NGCKKMRKLE IRDSPFGNAA
PsAFB2 FLYIGMYAEQ LEMLSIAFAG ESDKGMLYVL NGCKKLRKLE IRDCPFGDTA
AtAFB4 FSYIGEYGKL IRTLSVAFAG NSDKALRYVL EGCPKLQKLE IRDSPFGDVG
AtAFB5 FRYMGEYGKL VRTLSVAFAG DSDMALRHVL EGCPRLQKLE IRDSPFGDVA
PsAFB4 FEYIGRYGKL IRTLSVAFAG DTDTGLRYVL EGCPNLQKLE IRDSPFGDGA
Consistency *4*9*48774 776**9**** 68*468468* 5**4697*** ***6***848
560 570 580 590 600
AtTIR1 LLANASKLET MRSLWMSSCS VSFGACKLLG QKMP--KLNV EVIDERGAPD
PsTIR1a LLANAAKLET MRSLWMSSCH VSYGACKLLG LKLP--RLNV EVIDERGPPD
AtAFB1 LLEHAAKLET MRSLWMSSCF VSFGACKLLS QKMP--RLNV EVIDEH-PPE
PsTIR1b LLANAAKLET MRSLWMSSCP VSYGACKLLG QKMP--RLNV EVIDERGPPD
PsAFB6 LLAGLEKYES MRSLWMSSCR LTMNGCRFLA GEKP--RLNV EVMQE-----
AtAFB2 LLADVSKYET MRSLWMSSCE VTLSGCKRLA EKAPWLNVEI INENDNNRME
AtAFB3 LLADVGRYET MRSLWMSSCE VTLGGCKRLA QNSP--RLNV EIINENENNG
PsAFB2 LLTDVGKYET MRSLWMSSCE VTVGACKTLA KKMP--SLNV EIFNESE-QA
AtAFB4 LRSGMHRYSN MRFVWLSSCL ISRGGCRGVS HALP--NVVV EVFGADGDDD
AtAFB5 LRSGMHRYYN MRFVWMSACS LSKGCCKDIA RAMP--NLVV EVIGSDDDDD
PsAFB4 LRSGLHHYYN MRFLWMSSCK LTRQACQEVA RALP--HMVL EVINNGENAV
Consistency *665547667 **68*9*9*3 87366*8386 455*005859 8865633234

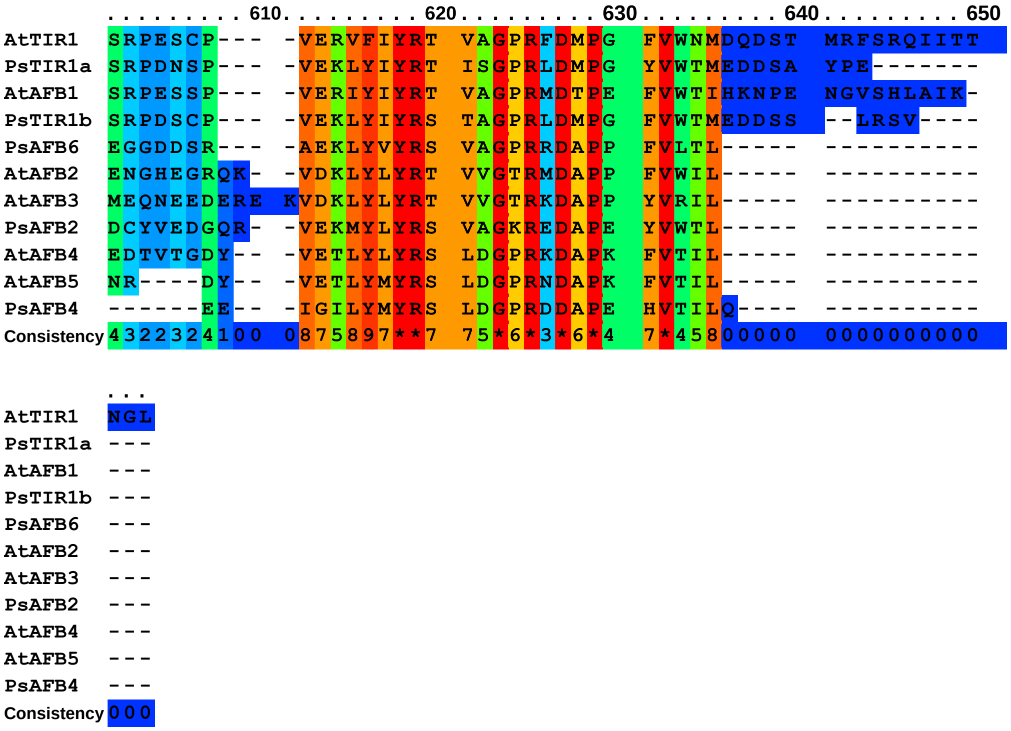

Supplemental Figure S4. Sequence alignment of pea and Arabidopsis auxin receptor proteins. The predicted pea (*Pisum sativum* L. cv. I_3 Alaska-type) TIR1/AFB auxin receptor proteins PsTIR1a, PsTIR1b, PsAFB2, PsAFB4, and PsAFB6 were aligned with the auxin receptor proteins of *Arabidopsis thaliana* (At) using the PRALINE multiple sequence alignment tool under default parameters. Color coding shows the amino acid conservation as shown in the legend at the beginning of the sequence alignment. Green, blue and red circles show the auxin, Aux/IAA and $InsP_6$ contacting amino acid residues of AtTIR1, respectively (Tan *et al.*, 2007). The blue dashed line denotes the F-box domain, and the solid black lines denote the leucine-rich repeat domains with respect to AtTIR1 (Dharmasiri *et al.*, 2005).

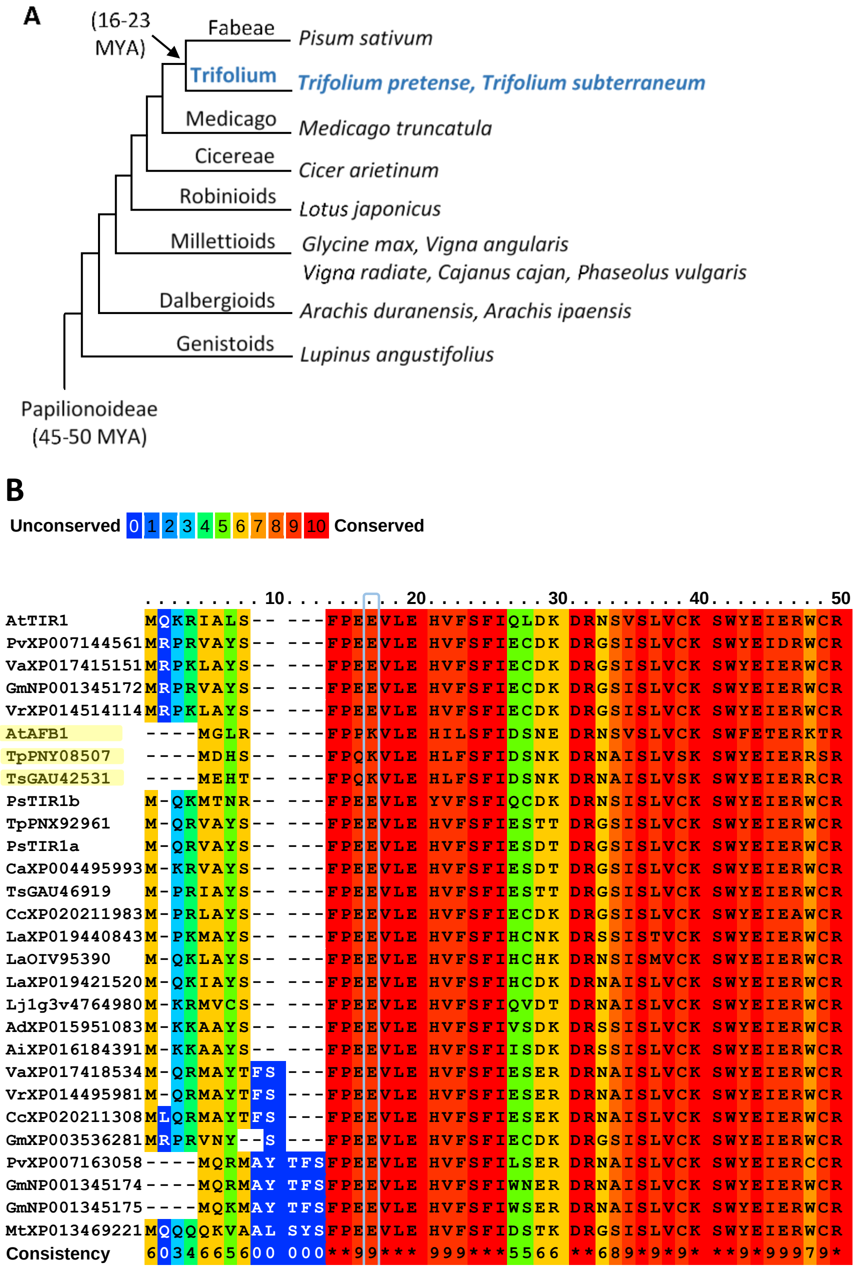

Supplemental Figure S5. Conservation of the F-box protein domain of TIR1 auxin receptor proteins in the Fabaceae subfamily Papilionoideae. (**A**) Taxonomic relationships within the Papilionoideae species based on those derived from Choi *et al.* (2004), Wojciechowski *et al.* (2004), Ellison *et al.* (2006), Lam *et al.* (2015) and Kaur *et al.* (2017). Branch lengths of the tree do not show phylogenetic distances. (**B**) Conservation of AtTIR1-E12 position (within the F-box domain) among the TIR1 clade of auxin receptors of *Arabidopsis thaliana* and selected species of the Papilionoideae. Species abbreviations are defined in the legend of Supplemental Figure S3. E-to-K substitution was found in the F-box domain of the TIR1b allele of species in the subgenera Trifolium (highlighted blue in A) after the divergence of the subgenera Trifolium and Fabeae.

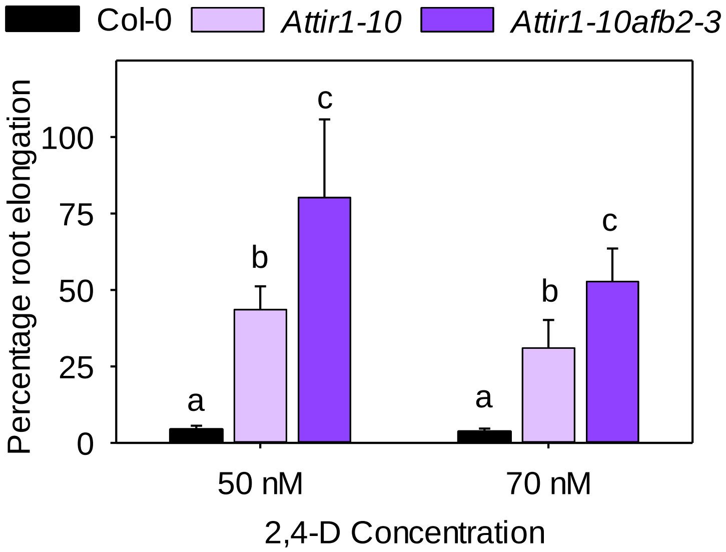

Supplemental Figure S6. Root elongation of Arabidopsis *tir1-10* and *tir1-10 afb2-3* mutant seedlings grown in the presence of 2,4-D. Four-day-old seedlings were transferred to media containing 0, 50, or 70 nM 2,4-D, and grown for three days before root length measurement. Root elongation of each genotype grown in media with 2,4-D is expressed as a percentage compared to the same genotype grown in media without 2,4-D. Data are means ± SD (n=8), Different letters denote significant differences within the same auxin concentration (One-way ANOVA with mean separation using the Holm-Sidak post-hoc test, $P<0.05$).

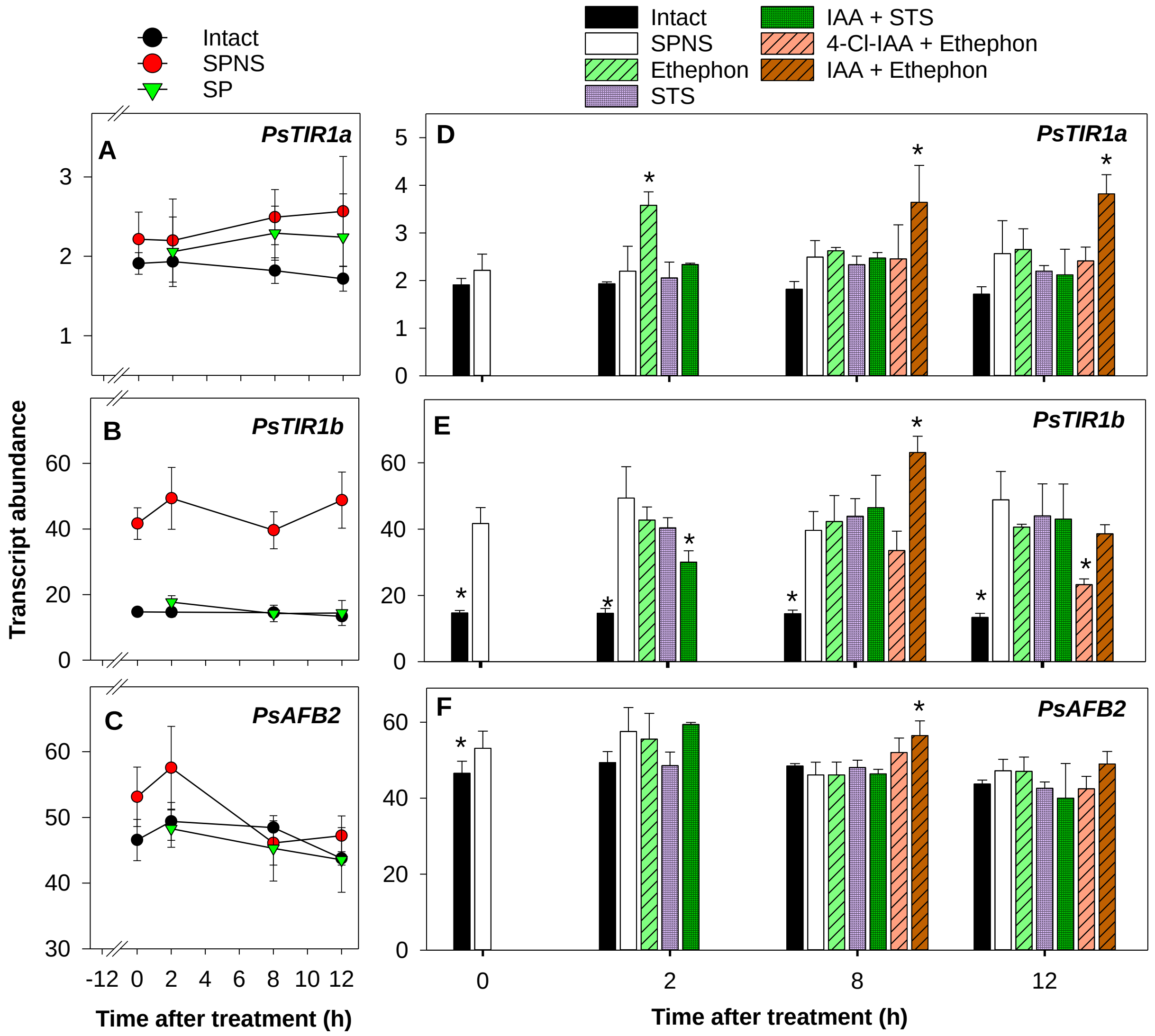

Supplemental Figure S7. Effect of seed removal and ethylene on the relative transcript abundance of the auxin receptors *PsTIR1a* (**A, D**), *PsTIR1b* (**B, E**), and *PsAFB2* (**C, F**) in the pea pericarp. Two days after anthesis pollinated fruits were either left intact, split (SP), or split and deseeded (SPNS). Deseeded pericarps were treated with the ethylene-releasing agent ethephon, ethylene-signaling inhibitor STS alone or with 4-Cl-IAA or IAA. When treated with auxin plus ethephon, ethephon was applied 90 min after the auxin treatment, and samples were collected based on the time after auxin treatment. Because of the delayed ethephon application, the auxin plus ethephon-treated pericarps were not studied at the 2 h time-point. STS was applied at pericarp splitting and deseeding (STS treatment), and IAA was applied 12 h after STS pretreatment (IAA plus STS treatment). SP and SPNS controls were treated with 0.1% aqueous Tween 80. All the samples were collected with respect to the time after hormone treatment. Data are means ± SD, n=3 to 8, with the exception of STS + IAA 2 h treatment, where n=2. * Means significantly different compared to the SPNS control within the time after treatment by one-way ANOVA and the Holm-Sidak post-hoc mean separation test, at P < 0.05.

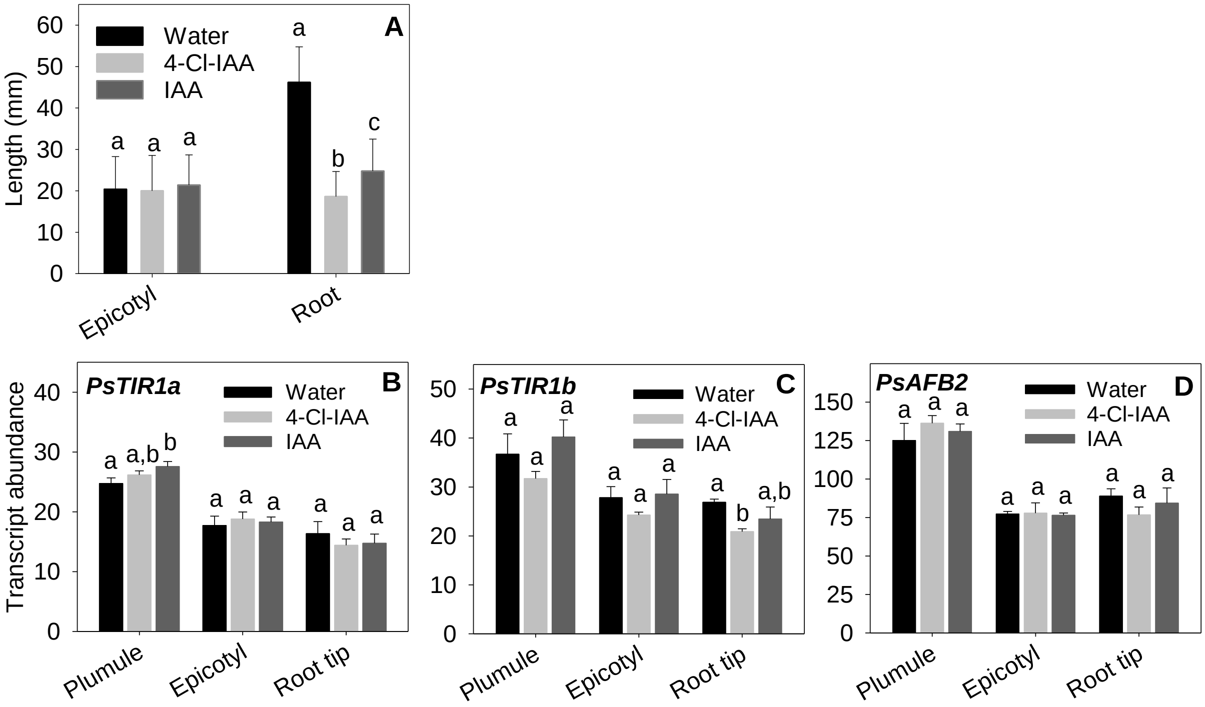

Supplemental Figure S8. Effect of IAA and 4-Cl-IAA on seedling growth and auxin-receptor abundance. Epicotyl and root length (A) and transcript abundance of *PsTIR1a* (B), *PsTIR1b* (C) and *PsAFB2* (D) in 4 DAI dark-grown seedlings. Two-day-old water-grown seedlings were selected for uniformity and transferred to 4-Cl-IAA (1 µM), IAA (1 µM) or water for an additional two days and harvested 4 DAI. Data are means ± SD (n=3, except in length measurements where n= 25-30). Different letters denote significant difference within tissue type by one-way ANOVA and the Holm-Sidak post-hoc mean separation test, $P<0.05$.

Supplemental Table S1. PCR primers used for gene cloning and verification. The primers listed were used for the amplification of the pea auxin receptors, Arabidopsis TIR1 promoter, or for the verification of Arabidopsis auxin receptor mutants.

**Target**	**Primer**	**Sequence**
PsTIR1a CDS	TIR1seq-Set2-FW	GGCGTTTCAGATCTAACTCGAACTCT
	TIR1seq-Set2-RV	GATCAGATTCCGTATCTAGTAGCTCAGCC
PsTIR1b CDS	PsAFB1-seqSet1-FW	ACACTTTAAGCTCGTTAATGGTGTC
	PsAFB1-seqSet2-RV	TGGCATTAAATATCTGCCACA
PsAFB2 CDS	PsAFB2-seqSet1-FW	TTCCATGAAAGTTGTAGAATTTGG
	PsAFB2-3′ UTRSet1-RV	ACTCAACATATTAATGAAGTGCCATC
PsAFB4 CDS	PsAFB4-seqSet1-FW	ATGAGAGAAAACCATCCTCCAAC
	PsAFB4-seqSet1-RV	CTTTGCGGCTTTCCATCAC
PsAFB6 CDS	PsAFB6- TR/FP	ATGGAACCACAAACCATGAATCCCAG
	PsAFB6-TR/RP	TCAGAGAGTGAGAACAAAAGGAGGTGC
AtTIR1 CDS	AtTIR1-sqF1	ATGCAGAAGCGAATAGCC
	AtTIR1-sqR1	TTATAATCCGTTAGTAGTAATGATTT
AtTIR1 Promoter	pAtT1-sqF1	TTGCAAACCCATCGAACGTT
	AtPM-TIR1set1-RV	CGTACCATGACTTGCACACC
tir1-10 (SALK_090445)	LP	CACGTGTCATCATCAGAATCG
	RP	ATTTCCCACCTCAGGAGATTC
afb2-3 (SALK_137151)	LP	TCAACGGTCAAGATCCATCTC
	RP	CTGCAATTAGCGGCAATAGAG
All SALK mutants	LBb1.3 (T-DNA boarder primer)	ATTTTGCCGATTTCGGAAC

Supplemental Table S2. Primers and probes used for qRT-PCR [a]

Gene	qRT-PCR Efficiency (%)	Primer /Probe	Sequence
PsTIR1a	94	Forward	TGCACTAGAGCGCCTGGTTA
		Reverse	TGTGGAGCCCCCCTAAGAA
		Probe	ACTTGCAGACGCTCAGGCTCAATCG
PsTIR1b	96	Forward	TGTCCTAACCTCCAGACTCTTCGT
		Reverse	GCTGAGGACATCGGCTAAGTAAGT
		Probe	ATCATGCTGCAGCCCTTGATAAACTGCC
PsAFB2	95	Forward	CATCCTCGATGCAACAAAACC
		Reverse	GACTGTACGATTGCCCCAAAA
		Probe	ACTCCGACACAATGCAGCCACTGGA
AtPP2AA3	99	Forward	AGCATGGCCGTATCATGTTCT
		Reverse	TGGCCAAAATGATGCAATCTC
		Probe	CACAACCGCTTGGTCGACTATCGGAAT
Ps18S rRNA	108	Forward	ACGTCCCTGCCCTTTGTACA
		Reverse	CACTTCACCGGACCATTCAAT
		Probe	ACCGCCCGTCGCTCCTAC CG

[a] The probes are 6-FAM fluorescent dye-labeled at 5′ end and double-quenched with Iowa Black FQ (IBFQ) quencher at the 3′ end and ZEN quencher in the middle, with one exception. For *Ps18S rRNA*, the probe is labelled with fluorescent reporter VIC at 5′ end and TAMRA quencher at the 3′ end.

SUPPLEMENTAL MATERIALS AND METHODS

Supplemental Protocol S1: Pea TIR1/AFB family sequence analysis and phylogenetic tree creation

Sequences of pea genes were verified by Sanger sequencing (performed at the Molecular Biology Service Unit, Department of Biological Sciences, University of Alberta). Sequence alignments to determine the conservation of amino acids important in auxin perception were done with the PRALINE sequence alignment program (Simossis and Heringa, 2003, 2005) under default settings.

For phylogenetic analysis, a neighbor-joining tree was created with the TIR1/AFB family proteins of pea, *A. thaliana*, apple (*Malus x domestica*), *Medicago truncatula* and the TIR1 orthologs of the legume species *Arachis duranensis* (Ad), *Arachis ipaensis* (Ai), *Cicer arietinum* (Ca), *Cajanus cajan* (Cc), *Glycine max* (Gm), *Lotus japonicus* (Lj), *Lupinus angustifolius* (La), *Phaseolus vulgaris* (Pv), *Trifolium pretense* (Tp), *Trifolium subterraneum* (Ts), *Vigna angularis* (Va) and *Vigna radiata* var. *radiata* (Vr). The tree was rooted with TIR1/AFB family proteins of the moss *Physcomitrella patens* (Pp). The Arabidopsis, apple and *P. patens* sequences were reported previously in Parry *et al.* (2009), Prigge *et al.* (2010) and Devoghalaere *et al.* (2012) respectively. All the other sequences were obtained from GenBank, and their GenBank accession numbers are given in the tree. Sequences were aligned using the MUSCLE program under the default settings. The tree was generated using the MEGA X program (Kumar *et al.*, 2018) with 1000 bootstrap replicates, Poisson correction model and partial deletion treatment with a site coverage cutoff set to 95%.

Supplemental Protocol S2: qRT-PCR assays

Each 25 µL reaction mixture contained 200 ng of total RNA (except for the quantification of 18S rRNA where each reaction mixture contained 100 pg of total RNA), 12.5 µL of 2X master mix, 0.6 µL of 40X MultiScribe/RT Enzyme Mix, 300 nM each of forward and reverse primers, 100 nM of probe and 3.4 µL of nuclease-free water. The thermal cycler conditions were: 48°C for 30 min (reverse transcription), 95°C for 10 min (DNA polymerase activation), 40

cycles of amplification at 95 ^{o}C for 15 sec and 60°C for 1 min. Each sample was run in duplicate, and the average of these two technical replicates was used as the sample value. A common, pooled total RNA sample was run on each qPCR plate as a control to correct plate to plate variation (Ayele *et al.*, 2006) as follows:

*Normalized Ct value of sample = (Ct value of the common sample in the standard plate / Ct value of the common sample in the sample plate) * Ct value of the sample*

The relative transcript abundance was calculated using the $2^{-\Delta Ct}$ method (Livak and Schmittgen, 2001), where ΔCt is the Ct difference of the sample being analyzed, and an arbitrary value equal to or greater than the highest assayed Ct value. The arbitrary Ct value was set at 25 for all genes when calculating their expression in pea tissues. qPCR efficiencies were analyzed using a dilution series of total RNA typically from 150 to 0.015 ng μL^{-1} (or 750 ng to 0.075 ng per reaction) with three technical replicates for each concentration. The 18S small subunit nuclear rRNA of pea was quantified as a loading control to estimate the variation of input total RNA of the samples. Primers and probe for the 18S rRNA control were designed by Ozga *et al*. (2003) with 5′ VIC as the fluorescent reporter of the probe and 3′ TAMRA as the quencher (Applied Biosystems). The coefficient of variation of the Ct values of 18S rRNA amplicon across all the samples was less than 3%. Therefore, the target amplicon expression values were not normalized to the 18S signal (Livak and Schmittgen, 2001; Ozga *et al.*, 2009).

Pea auxin receptor transcript levels in transgenic Arabidopsis plants were analyzed as described above with following exceptions. Arabidopsis protein phosphatase 2A subunit A3 (AtPP2AA3; Arabidopsis Gene ID: AT1G13320) was used as a loading control to estimate the variation of input total RNA of the samples (Czechowski *et al.*, 2005; Wang *et al.*, 2014). The arbitrary Ct value was set at 35 for all genes for the calculation of relative transcript abundance.

Supplemental Protocol S3: Arabidopsis transgenic plant creation and root growth analysis

Transformation

Recombinant plasmids carrying *pAtTIR1*::*cPsTIR1a*, *pAtTIR1*::*cPsTIR1b*, *pAtTIR1*::*cPsAFB2* or *pAtTIR1*::*cAtTIR1* were transformed into *Agrobacterium tumefaciens* strain GV3101 by electroporation. For Arabidopsis transformation, the floral-dip transformation method was used as described in Zhang *et al.* (2006). Progenies containing the transgene were

selected by growing the seeds of floral-dipped plants in hygromycin and verified by PCR. A hygromycin segregation assay was used with seeds obtained from T2 plants to identify homozygous transgenic lines that segregated consistent with single gene insertion events.

Hygromycin selection

The method for hygromycin selection of transformed plants was adapted from Bent (2006). Seeds were sterilized with 70% ethanol (2 min), and then 2% NaOCl containing 0.1% Tween 80 (10 min). Sterilized seeds were spread on plates containing half-strength Murashige and Skoog (MS) medium containing 25 mg L^{-1} hygromycin. The plates were kept in the dark at 4°C for four days, then exposed to light to induce germination at ~22°C for 6 to 8 h, and subsequently in continuous dark at ~22°C for four days. At the end of the fourth day, the hygromycin-resistant transformants could be differentiated from non-transformants by their elongated hypocotyls and closed cotyledons (Bent, 2006).

Seeds (T_2 generation) from each independent T_1 transformant line were germinated, and the seedlings were grown separately to obtain 12 T_2 plants from each line. A portion of seeds (about 30 to 60) from those plants (T_3 seeds) was used to repeat the hygromycin selection assay to see if the parent plant was homozygous for the transgene construct. Independent transgenic lines showing an approximately 1:2:1 (homozygous: hemizygous: wild-type) segregation ratio for hygromycin resistance were considered to contain single insertions. The only exception to the above selection criteria was with *pAtTIR1::cPsTIR1a* in *Attir1-10*, where several T1 plants were pooled instead of collecting seeds separately from each plant. The T2 seedlings from this seed lot did not segregate for hygromycin resistance (all were hygromycin resistant).

Root growth assay

Two independent transgenic lines per gene construct (T4 generation) that were homozygous for the transgene insert, segregated on selection media consistent with a single gene insertion event, and expressed the transgene at reliable levels (Supplemental Fig. S1 B-D), were selected for root growth assays. Two independent homozygous transgenic lines (T4 generation) per gene construct were assessed for root elongation. For root growth assays with *PsTIR1a* and *PsTIR1b*, the final auxin concentrations were 50 or 70 nM for 2,4-D, and 400 or 800 nM for IAA and 4-Cl-IAA. For root growth assays with *PsAFB2*, final 2,4-D concentrations were 70 and 90

nM. Root elongation of each genotype in auxin medium is expressed as a percentage of the same genotype in the medium without auxin

Surface sterilized Arabidopsis seeds were placed on 1% aqueous (w/v) agar plates (100 x 100 x 15 mm square plates with grid) containing half-strength MS and 1% (w/v) sucrose. The plates were kept in the dark at 4°C for four days for seed stratification and then moved to a growth chamber maintained at 22°C with continuous fluorescent light. Four-day-old uniform-size seedlings were transferred to square plates containing the same medium as above but with or without auxins for three days.

Root elongation assays with 2,4-D in the media were performed in plates under fluorescent light (average photosynthetic flux density; 155 ± 20 µmol m^{-2} s^{-1}); assays with IAA or 4-Cl-IAA in the media were performed in plates under yellow plastic film (which filtered out the wavelengths between 350 nm to 500 nm with a maximum absorbance at 430 nm) to prevent the photodegradation of auxin in the growth media (Stasinopoulos and Hangarter, 1990). The average photosynthetic flux density for the plates covered with the yellow plastic film was about 100 ± 10 µmol m^{-2} s^{-1}. Each plate contained two seedlings each (considered as technical replicates during analysis) of all the relevant transgenic or mutant lines with appropriate controls used for comparison. The total number of seedlings per root growth assay plate was always maintained at 12. Seedling root tip positions were marked on the day of transfer to the new plates and were remarked after three days. Plates were photographed using a Canon-PC1438 camera positioned horizontally to the vertically kept plates. Root elongation was measured using ImageJ software (1.49v, Abràmoff *et al*., 2004) as described in Doerner (2008).

Supplemental Protocol S4: GUS staining and quantification of GUS enzyme activity

DR5-driven expression of the *GUS* marker gene was monitored over development in prepollinated (-2 DAA) and pollinated fruits at 0, 3, 5, 8 and 10 DAA in *DR5::GUS* line: ADR5P-R24A. The GUS expression patterns were verified with a second independent line of *DR5::GUS* plants, ADR5P-R22A. Two DAA deseeded (SPNS) pericarps were treated with auxin (IAA or 4-Cl-IAA at 50 µM in 0.1% aqueous Tween 80) or 0.1% aqueous Tween 80 12 h after deseeding, and pericarps were collected at 2, 8 and 12 h after treatment for GUS staining and measuring GUS enzyme activity.

GUS staining

For GUS staining, the pericarp sections were submerged in 12- or 24-well tissue culture plates containing GUS staining solution [1 mM 5-Bromo-4-chloro-3-indolyl β-D-glucuronide (X-Gluc), 100 mM sodium-phosphate buffer (pH 7), 0.5 mM potassium ferrocyanide, 0.5 mM potassium ferricyanide, 0.1% Triton X100 and 10 mM EDTA]. Samples were then placed in a vacuum desiccator for 30 min and subsequently incubated in the dark at 37°C. After 12 h, samples were washed approximately three times with 70% ethanol before taking micrographs using a Zeiss SteREO Discovery V8 (Carl Zeiss MicroImaging GmbH, Jena, Germany) or Olympus SZ61 (Olympus Corporation, Tokyo, Japan) stereo microscope.

GUS enzyme activity analysis

Tissues were ground by bead-beating and mixed with 150 µL of GUS extraction buffer [50 mM sodium phosphate buffer (pH 7.0), 10 mM Na_2EDTA, 0.1% sodium lauroyl sarcosinate, 0.1% Triton X-100, l0 mM β-mercaptoethanol]. The mixture was spun down to separate out the supernatant (tissue extract), which was frozen in liquid nitrogen and stored at -80°C until analysis.

Prior to the MUG assay, tissue extracts from the IAA- and 4-Cl-IAA-treated samples were diluted 3- and 5-fold, respectively, due to high GUS enzyme activity in those samples. Twenty microliters of the tissue extract was mixed with 200 µL of pre-warmed assay buffer (1 mM MUG in extraction buffer) and incubated in a 37°C shaking water bath. At pre-determined time points (15, 30 and 45 min for developmental assays; 5, 10 and 15 min for hormone treatment assays), 60 µL aliquots were taken out from the reaction solution and mixed with 540 µL of stop buffer (0.2 M Na_2CO_3). The amount of the end product, 4-methylumbelliferone (4-MU), produced during the reaction period was analyzed by measuring fluorescence using a SpectraMax M3 Multi-Mode Microplate Reader with excitation, emission, and cut-off bandwidths set to 365 nm, 455 nm, and 435 nm, respectively. A nine-point dilution series of a 4-MU (Sigma) standard with concentrations ranging from 15.625 to 4000 nM was run on each plate. A five-point range appropriate for the fluorescence intensity of samples was selected for the creation of the calibration curve. Each sample was analyzed twice, and the average was taken for the calculations.

The MUG assay data was normalized to the total protein content of the sample, measured using the Bio-Rad Protein Assay (Cervera, 2004). Four technical replicates were analyzed per sample. After 10 min, sample absorbance was measured at 595 nm in a SpectraMax M3 Multi-

Mode Microplate Reader. A three-point dilution series of bovine serum albumin (ranging from 0.06- 0.24 mg/mL) was used to construct a calibration curve.

Supplemental Protocol S5: Hormonal analysis

Pea fruits from plants expressing the DR5::GUS transgene (ADR5P-R22A) were used for the hormonal analysis. The following is the protocol used by the National Research Council, Saskatoon, Canada (NRCC SK) for free and conjugated IAA analysis.

Chemicals and calibration curves

The IAA conjugate IAA-Glu was synthesized and prepared at the NRCC SK. IAA-Leu, IAA-Ala, IAA-Asp, and IAA were purchased from Sigma–Aldrich. Deuterated forms of the hormones, which were used as internal standards, d_3-IAA-Leu, d_3-IAA-Ala, d_3-IAA-Asp, d_3-IAA-Glu, and 1,2,3,4-$^{13}C_4$-IBA (unpublished), were synthesized and prepared at NRCC SK according to Abrams *et al*. (2003) and Zaharia *et al*. (2005). d_5-IAA was purchased from Cambridge Isotope Laboratories (Andover, MA). Calibration curves were created for all compounds of interest. Quality control samples were run along with the tissue samples.

Instrumentation

Analysis was performed on a UPLC/ESI-MS/MS utilizing a Waters ACQUITY UPLC system, equipped with a binary solvent delivery manager and a sample manager coupled to a Waters Micromass Quattro Premier XE quadrupole tandem mass spectrometer via a Z-spray interface. MassLynx and QuanLynx (Micromass, Manchester, UK) were used for data acquisition and data analysis.

Extraction and purification

An acidified aliquot (100 μL) containing all the internal standards, each at a concentration of 0.2 ng μL^{-1}, was added to 1 mL weighed homogenized liquid sample containing approximately 50 mg dry weight of tissue. To this sample, 3 mL of isopropanol: water: glacial acetic acid (80:19:1, v/v/v) were added, and the samples were agitated in the dark for 14-16 h at 4 ºC. Samples were then centrifuged and the supernatant was isolated and dried on a Büchi Syncore Polyvap (Büchi, Switzerland). The dried sample residue was reconstituted in 100 μL acidified methanol, adjusted to 1 mL with acidified water, and then

partitioned against 2 mL hexane. After 30 min, the aqueous layer was isolated and dried as above. Dry samples were reconstituted in 800 μL acidified methanol and adjusted to 1 mL with acidified water. The reconstituted samples were passed through equilibrated Sep-Pak C18 cartridges (Waters, Mississauga, ON, Canada), and then the final eluate was split in two equal portions. One portion (#1) was dried completely (and stored) while the other portion was dried down to the aqueous phase on a LABCONCO centrivap concentrator (Labconco Corporation, Kansas City, MO, USA). The second portion was partitioned against ethyl acetate (2 mL) and further purified using an Oasis WAX cartridge (Waters, Mississauga, ON, Canada). Fraction (#2) was eluted with 2 mL acetonitrile: water (80:20, v/v) and then dried on a centrivap as described above. An internal standard blank was prepared with 100 μL of the deuterated internal standards mixture. A quality control standard was prepared by adding 100 μL of a mixture containing all the analytes of interest, each at a concentration of 0.2 ng μL^{-1}, to 100 μL of the internal standard mix. Finally, fractions #1 and #2, blanks, and quality controls were reconstituted in a solution of 40% methanol (v/v), containing 0.5% acetic acid and 0.1 ng μL^{-1} of each of the recovery standards.

Hormone quantification by HPLC-ESI-MS/MS

The procedure for quantification of auxins in plant tissue was performed as described in Lulsdorf *et al*. (2013). Samples were injected onto an ACQUITY UPLC® HSS C18 SB column (2.1x100 mm, 1.8 μm) with an in-line filter and separated by a gradient elution of water containing 0.02% formic acid against an increasing percentage of a mixture of acetonitrile and methanol (50:50, v/v).

Briefly, the analysis utilizes the Multiple Reaction Monitoring (MRM) function of the MassLynx v4.1 (Waters Inc) control software. The resulting chromatographic traces are quantified off-line by the QuanLynx v4.1 software (Waters Inc) wherein each trace is integrated and the resulting ratio of signals (non-deuterated/internal standard) is compared with a previously constructed calibration curve to yield the amount of analyte present (ng per sample). Calibration curves were generated from the MRM signals obtained from standard solutions based on the ratio of the chromatographic peak area for each analyte to that of the corresponding internal standard, as described by Ross *et al*. (2004). The quality control samples, internal standard blanks and solvent blanks were also prepared and analyzed along with each batch of tissue samples.

SUPPLEMENTAL REFERENCES

Abràmoff MD, Magalhães PJ, Ram SJ. 2004. Image processing with ImageJ. Biophotonics International **11**, 36–42.

Abrams SR, Nelson K, Ambrose SJ. 2003. Deuterated abscisic acid analogs for mass spectrometry and metabolism studies. Journal of Labelled Compounds and Radiopharmaceuticals **46**, 273–283.

Ayele BT, Ozga JA, Kurepin L V, Reinecke DM. 2006. Developmental and embryo axis regulation of gibberellin biosynthesis during germination and young seedling growth of pea. Plant Physiology **142**, 1267–1281.

Bent A. 2006. *Arabidopsis thaliana* floral dip transformation method. In: Wang K, ed. Methods in Molecular Biology. Agrobacterium Protocols SE - 7. Humana Press, 87–104.

Cervera M. 2004. Histochemical and fluorometric assays for uidA (GUS) gene detection. Transgenic Plants: Methods and Protocols. Springer, 203–213.

Choi H-K, Mun J-H, Kim D-J, *et al.* 2004. Estimating genome conservation between crop and model legume species. Proceedings of the National Academy of Sciences USA **101**, 15289–15294.

Czechowski T, Stitt M, Altmann T, Udvardi MK, Scheible W-R. 2005. Genome-wide identification and testing of superior reference genes for transcript normalization in Arabidopsis. Plant Physiology **139**, 5–17.

Devoghalaere F, Doucen T, Guitton B, *et al.* 2012. A genomics approach to understanding the role of auxin in apple (*Malus x domestica*) fruit size control. BMC Plant Biology DOI: 10.1186/1471-2229-12-7.

Dharmasiri N, Dharmasiri S, Estelle M. 2005. The F-box protein TIR1 is an auxin receptor. Nature **435**, 441–445.

Doerner P. 2008. Phenotypic analysis of Arabidopsis mutants: Quantitative analysis of root growth. Cold Spring Harbor Protocols DOI: 10.1101/pdb.prot4960

Ellison NW, Liston A, Steiner JJ, Williams WM, Taylor NL. 2006. Molecular phylogenetics of the clover genus (Trifolium—Leguminosae). Molecular Phylogenetics and Evolution **39**, 688–705.

Jayasinghege CPA, Ozga JA, Waduthanthri KD, Reinecke DM. 2017. Regulation of ethylene-related gene expression by indole-3-acetic acid and 4-chloroindole-3-acetic acid in relation to pea fruit and seed development. Journal of Experimental Botany **68**, 4137–4151.

Kaur P, Bayer PE, Milec Z, *et al.* 2017. An advanced reference genome of *Trifolium subterraneum* L. reveals genes related to agronomic performance. Plant Biotechnology Journal **15**, 1034–1046.

Kumar S, Stecher G, Li M, Knyaz C, Tamura K. 2018. MEGA X: Molecular evolutionary

genetics analysis across computing platforms. Molecular Biology and Evolution **35**, 1547–1549.

Lam HK, McAdam, Scott AM, McAdam EL, Ross JJ. 2015. Evidence that chlorinated auxin is restricted to the Fabaceae but not to the Fabeae. Plant Physiology **168**, 798-803.

Livak KJ, Schmittgen TD. 2001. Analysis of relative gene expression data using real-time quantitative PCR and the 2(-Delta Delta C(T)) Method. Methods **25**, 402–8.

Lulsdorf MM, Yuan HY, Slater SMH, Vandenberg A, Han X, Zaharia LI, Abrams SR. 2013. Endogenous hormone profiles during early seed development of *C. arietinum* and *C. anatolicum*. Plant Growth Regulation **71**, 191–198.

Ozga JA, Reinecke DM, Ayele BT, Ngo P, Nadeau C, Wickramarathna AD. 2009. Developmental and hormonal regulation of gibberellin biosynthesis and catabolism in pea fruit. Plant Physiology **150**, 448–462.

Ozga JA, Yu J, Reinecke DM. 2003. Pollination-, development-, and auxin-specific regulation of gibberellin 3β-hydroxylase gene expression in pea fruit and seeds. Plant Physiology **131**, 1137–1146.

Parry G, Calderon-Villalobos LI, Prigge M, Peret B, Dharmasiri S, Itoh H, Lechner E, Gray WM, Bennett M, Estelle M. 2009. Complex regulation of the TIR1/AFB family of auxin receptors. Proceedings of the National Academy of Sciences USA **106**, 22540–22545.

Prigge MJ, Lavy M, Ashton NW, Estelle M. 2010. *Physcomitrella patens* auxin-resistant mutants affect conserved elements of an auxin-signaling pathway. Current Biology **20**, 1907–1912.

Ross ARS, Ambrose SJ, Cutler AJ, Feurtado JA, Kermode AR, Nelson K, Zhou R, Abrams SR. 2004. Determination of endogenous and supplied deuterated abscisic acid in plant tissues by high-performance liquid chromatography-electrospray ionization tandem mass spectrometry with multiple reaction monitoring. Analytical Biochemistry **329**, 324–333.

Simossis VA, Heringa J. 2003. The PRALINE online server: optimising progressive multiple alignment on the web. Computational Biology and Chemistry **27**, 511–519.

Simossis VA, Heringa J. 2005. PRALINE: A multiple sequence alignment toolbox that integrates homology-extended and secondary structure information. Nucleic Acids Research **33**, W289–W294.

Stasinopoulos TC, Hangarter RP. 1990. Preventing photochemistry in culture media by long-pass light filters alters growth of cultured tissues. Plant Physiology **93**, 1365–1369.

Tan X, Calderon-Villalobos LIA, Sharon M, Zheng C, Robinson C V, Estelle M, Zheng N. 2007. Mechanism of auxin perception by the TIR1 ubiquitin ligase. Nature **446**, 640–645.

Wang H, Wang J, Jiang J, Chen S, Guan Z, Liao Y, Chen F. 2014. Reference genes for normalizing transcription in diploid and tetraploid Arabidopsis. Scientific Reports **4**, 6781.

Wojciechowski MF, Lavin M, Sanderson MJ. 2004. A phylogeny of legumes (Leguminosae) based on analysis of the plastid matK gene resolves many well-supported subclades within the family. American Journal of Botany **91**, 1846–1862.

Zaharia LI, Galka MM, Ambrose SJ, Abrams SR. 2005. Preparation of deuterated abscisic acid metabolites for use in mass spectrometry and feeding studies. Journal of Labelled Compounds and Radiopharmaceuticals **48**, 435–445.

Zhang X, Henriques R, Lin S-S, Niu Q-W, Chua N-H. 2006. Agrobacterium-mediated transformation of *Arabidopsis thaliana* using the floral dip method. Nature Protocols **1**, 641–646.
